# Chemical constituents of *Lycium barbarum* leaves and their anti-rheumatoid arthritis activity in vitro

**DOI:** 10.1007/s13659-025-00516-9

**Published:** 2025-05-30

**Authors:** Zi-Jiao Wang, Bang-Yin Tan, Yun Zhao, Chang-Bin Wang, Yun-Li Zhao, Xiao-Dong Luo

**Affiliations:** 1 State Key Laboratory of Phytochemistry and Plant Resources in West China, Kunming Institute of Botany, Chinese Academy of Sciences, Kunming, 650201 P. R. China; 2 Yunnan Characteristic Plant Extraction Laboratory Co. Ltd, Key Laboratory of Medicinal Chemistry for Natural Resource, Ministry of Education, Yunnan Key Laboratory of Research and Development for Natural Products, School of Pharmacy, School of Chemical Science and Technology, Yunnan University, Southwest United Graduate School, Kunming, 650091 P. R. China; 3 University of Chinese Academy of Sciences, Beijing, 100049 P. R. China

**Keywords:** *Lycium barbarum* leaves, Chemical constituents, Anti-rheumatoid arthritis

## Abstract

Two new together with 32 known compounds were isolated from the leaves of *Lycium barbarum*. Their structures were elucidated using 1D and 2D NMR, HRESIMS, and ECD spectroscopic techniques. Compounds **1–34** were evaluated for their anti-rheumatoid arthritis activities in a lipopolysaccharide (LPS)-induced MH7A cells inflammatory model. As a result, compounds **1–3**, **6**, **8**, **10**, **14**, **17–19**, **29** and **31** inhibited the activity of lactate dehydrogenase (LDH) and nitric oxide (NO) at concentrations 20 μM. Among them, compound **1** showed the best effectiveness, with inhibition rates of 46.7% for NO and 32.8% for LDH.

**Graphical Abstract:**

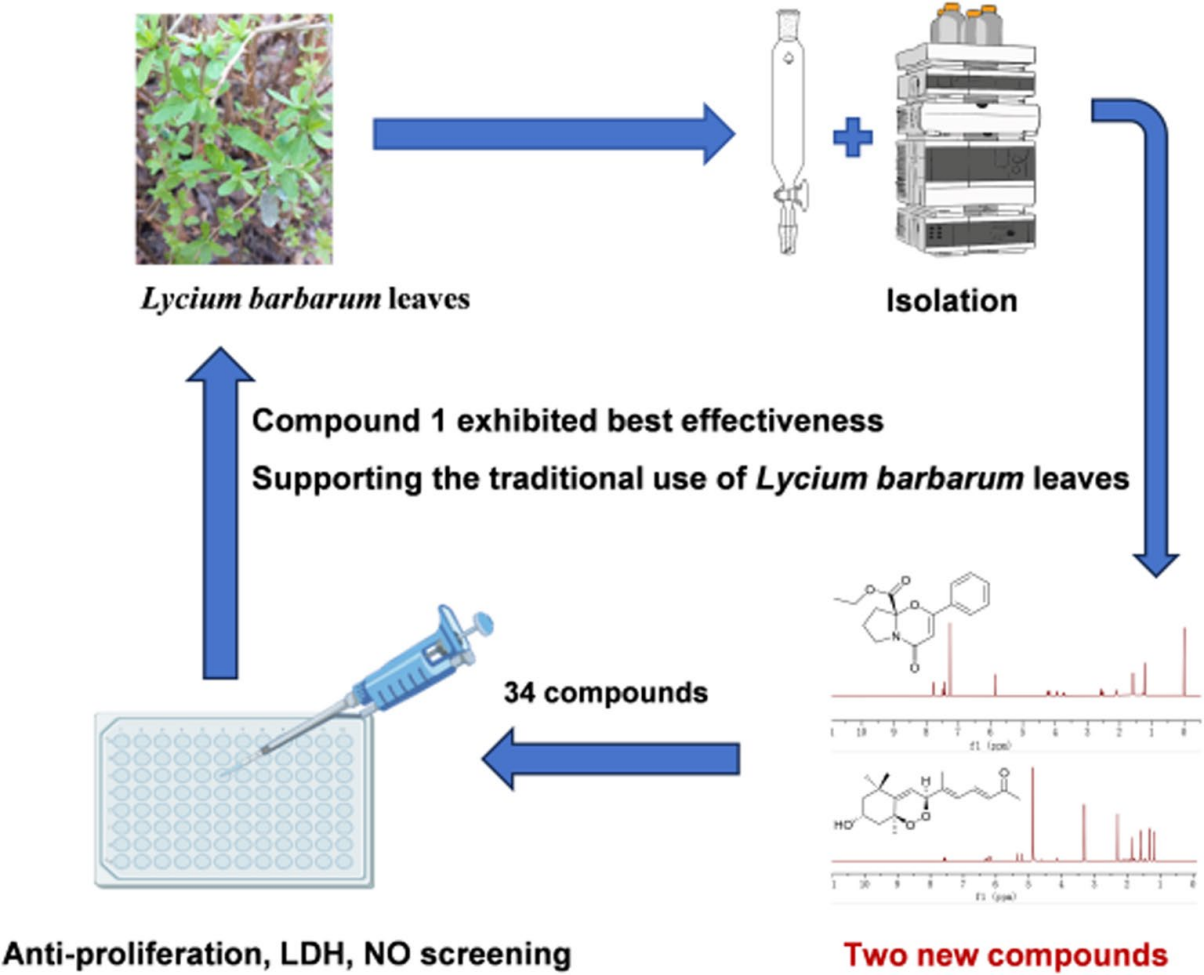

**Supplementary Information:**

The online version contains supplementary material available at 10.1007/s13659-025-00516-9.

## Introduction

*Lycium barbarum* L., a deciduous shrub from the Solanaceae family, is a renowned traditional plant for both medicine and food [1]. The leaves of *L. barbarum* were proven to possess various biological effects such as boosting immunity, reducing heat, alleviating rheumatic pain, quenching thirst, promoting saliva secretion, and improving eyesight [2]. In Asian countries, the leaves served as functional vegetables, commonly used in soup making, stir-frying, and as herbal teas [3].

Rheumatoid arthritis (RA), characterized by persistent inflammation and abnormal proliferation of fibroblast-like synoviocytes (FLS), was a chronic inflammatory condition that could result in joint destruction and disability [4]. The treatment of RA included glucocorticoids, disease-modifying antirheumatic drugs, nonsteroidal anti-inflammatory drugs and biologics [5]. However, these medications were expensive and came with serious side effects [6]. Then, it was essential to explore natural products for anti-RA drugs that were effective and had low toxicity. Folk applications suggested the therapeutic effects of *L. barbarum* leaves on rheumatoid arthritis, but its bioactive compounds remained unknown.

## Results and discussion

### Structural elucidation of compounds

**Fig. 1:**
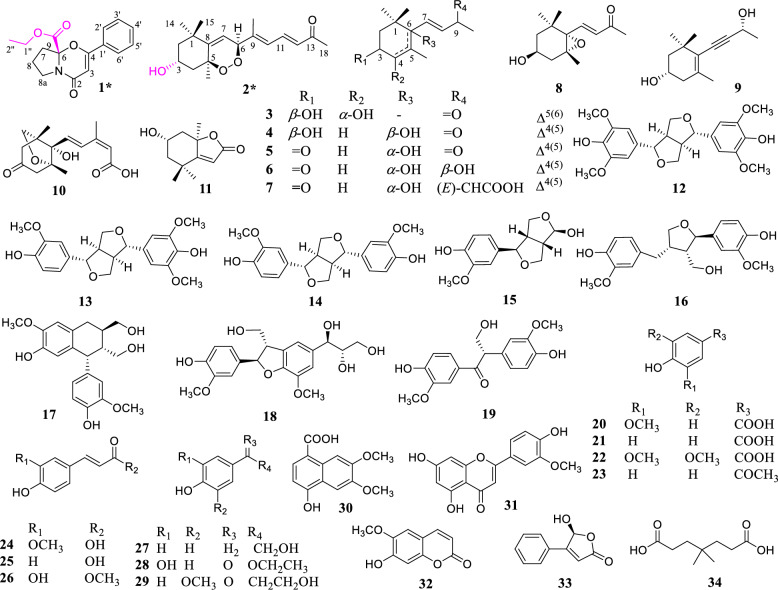
Structures of **1–34**

A total of 34 compounds were isolated from leaves of *L. barbarum*, including one alkaloid (**1**), 10 terpenoids (**2–11**), 8 lignans (**12**−**19**), 11 phenolic acids (**20**−**30**), and four other compounds (**31**−**34**) with compounds (**1** and **2**) being newly discovered (Fig. 1). Compounds **4** and **8–19** were first isolated from *L. barbarum* leaves.

**Fig. 2:**
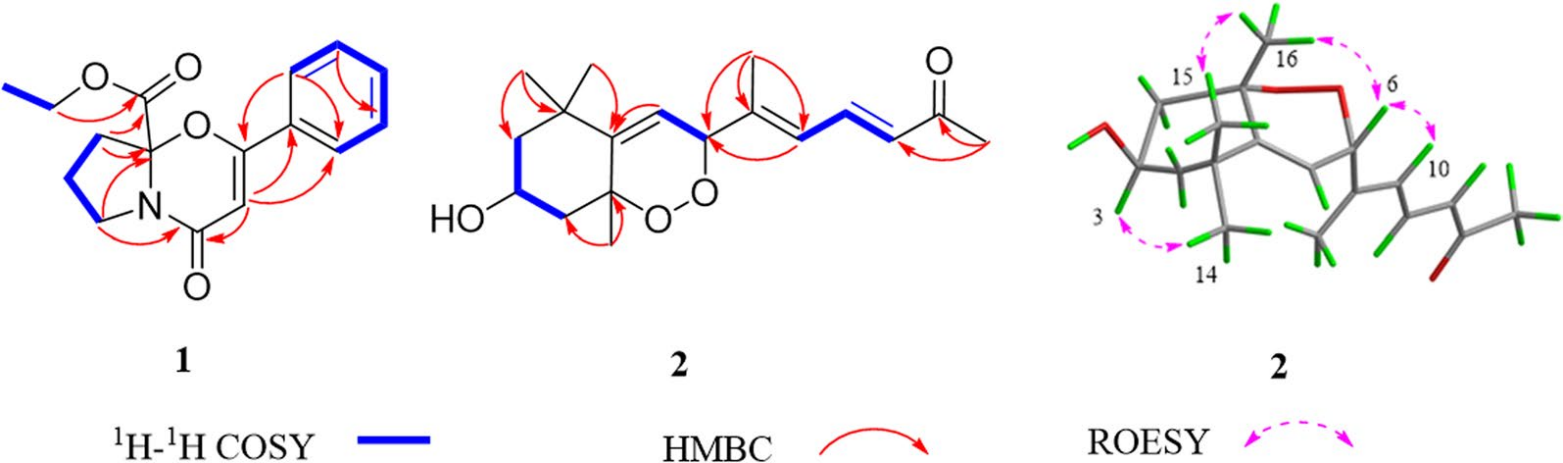
The key ^1^H−^1^H COSY and HMBC correlations of compounds **1** and **2**

**Fig. 3:**
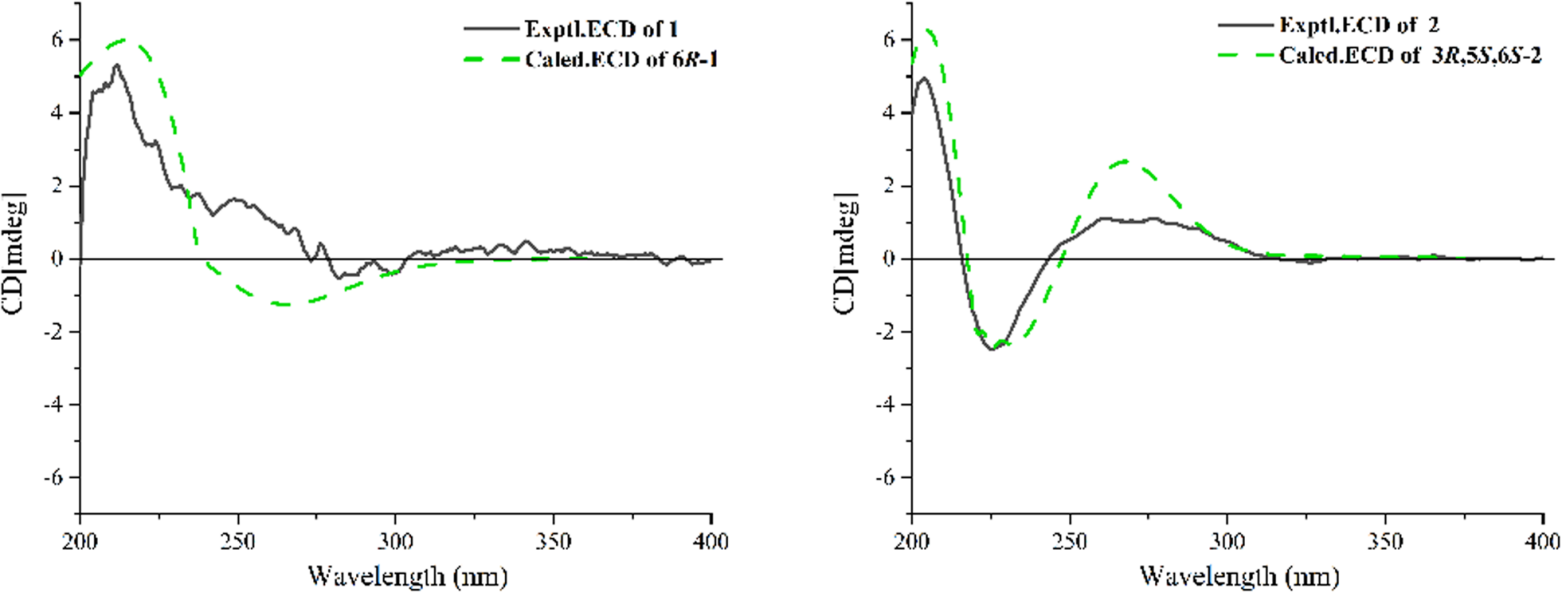
The experimental and calculated ECD spectra of compounds **1** and **2**

The molecular formula of lycibarin A (**1**) was assumed to be C_16_H_17_NO_4_ based on HRESIMS peak at m/z 288.1221 [M + H]^+^(calcd for C_16_H_17_NO_4_^+^, 288.1230) and the ^13^C NMR spectrum, requiring 9 degrees unsaturation. Its IR spectrum displayed characteristic bands assignable to the carbonyl group (1766 cm^−1^), and olefinic (1652 cm^−1^). The 1D and 2D NMR spectroscopic data suggested that **1** was structurally similar to 6,7,8,8a-tetrahydro-2-phenyl-4*H*-pyrrolo[2, 1-b][1, 3]oxazin-4-one [7] with the notable difference being the presence of an additional ethyl acetate fragment in **1**. Meanwhile, the COSY correlation of *δ*_H_ 1.23 (H-2'') / 4.23 (H-1''*α*), and the HMBC correlation between *δ*_H_ 4.23 (H-1''*α*) and *δ*_C_ 170.5 (C-9) supported the presence of ethyl acetate group, while the ethyl acetate substitution at C-6 was indicated by the HMBC correlations of *δ*_H_ 2.55 (H-7) with *δ*_C_ 94.8 (C-6) and 170.5 (C-9). Then the structure of **1** was elucidated as illustrated in Fig. 2, and its chiral carbon was deduced to be 6*R* by comparing the experimental and calculated electronic circular dichroism (ECD) spectra (Fig. 3).

Compound** 2** possessed a molecular formula of C_18_H_26_O_4_ by its ( +)-HRESIMS data at m/z 329.1698 [M + Na]^+^ (calcd for C_18_H_26_O_4_Na, 329.1723). Detailed analysis of the ^1^H and ^13^C NMR spectral data (Table 1) of **2** showed high similarity to those of 5,8-endoperoxy-2,3-dihydro-*β*-apocarotene-13-one [8]. The obvious differences between the two compounds were **2** with oxymethine signal at C-3 (*δ*_C_ 68.1) instead of methylene signal (*δ*_C_ 18.6) in 5,8-endoperoxy-2,3-dihydro-*β*-apocarotene-13-one, which assumed a hydroxylated derivative of 5,8-endoperoxy-2,3-dihydro-*β*-apocarotene-13-one for **2**. Meanwhile, the COSY correlation (Fig. 2) of *δ*_H_ 4.14 (H-3) / 1.78 (H_2_-2*α*) supported the hydroxyl substitution at C-3. The large coupling constant (*J*_H-11/H-12_ = 15.4 Hz) suggested *E* configuration for ∆^11,12^, and the *E* configuration of ∆^9,10^ was supported by the NOE correlation of *δ*_H_ 5.20 (H-6) / 6.31 (H-10) in its ROESY spectrum. Additionally, NOE correlations of *δ*_H_ 5.20 (H-6) / 1.60 (H-16) and *δ*_H_ 1.60 (H-16) / 1.33 (H-15) indicated their *syn* orientation and temporarily assigned the *α*-orientation, while the *β*-configuration of H-3 was elucidated by the NOE correlation between *δ*_H_ 4.14 (H-3) / 1.18 (H_3_-14) (Fig. 2). Moreover, the calculated ECD spectrum of (3R*, 5S*, 6S*) configuration was matched the experimental spectrum of **2**, which assigned its absolute configuration (Fig. 3).

**Table 1:** The ^1^H NMR (600 MHz) and ^13^C NMR (150 MHz) spectral data of compounds **1** and **2** (*δ* in ppm and* J* in Hz)

NO	1^a^		2^b^	
	***δ***_**H**_ (mult, *J*)	***δ***_**C**_	***δ***_**H**_ (mult, *J*)	***δ***_**C**_
1				34.9, s
2*α*		161.6, s	1.78, dd (14.0, 7.4)	48.7, t
2*β*			1.48, dd (14.0, 3.5)	
3	5.85, s	98.5, d	4.14, m	68.1, d
4*α*		163.7, s	2.10, dd (13.5, 3.8)	48.9, t
4*β*			1.95, dd (13.5, 4.4)	
5				89.5, s
6		94.8, s	5.20, br s	88.6, d
7	2.55, m	36.8, t	5.35, br s	120.2, d
8	2.10, m	21.2, t		156.3, s
8a*α*	3.93, dt (11.0, 7.0)	45.2, t		
8a*β*	3.73, dt (11.0, 7.0)	-		
9	-	170.5, s		151.2 s
10			6.31, d (11.4)	125.3, d
11			7.57, dd (15.4, 11.4)	141.2, d
12			6.19, d (15.4)	131.5, d
13				201.8, s
14			1.18, s	31.9, q
15			1.33, s	29.7, q
16			1.60 s	29.6, q
17			1.86, s	13.5, q
18			2.30, s	27.4, q
1'	-	131.5, s		
2'/6'	7.78, d (7.6)	127.2, d		
3'/5'	7.44, t (7.6)	128.6, d		
4'	7.48, t (7.6)	131.4, d		
1''*α*	4.23, dq (10.8, 7.2)	62.4, t		
1''*β*	4.18, dq (10.8, 7.2)			
2''	1.23, t (7.2)	14.0, q		

a: in CDCl_3_

b: in CD_3_OD

The 32 known compounds (**3**−**34**) were identified as (−)-(3*S*,4*S*)-eucomehastigmane B (**3**) [9], *cis*-3,6-dihydroxy-*α*-ionone (**4**) [10], ( +)-dehydrovomifoliol (**5**) [11], vomifoliol (**6**) [12], *trans, trans*-abscisic acid (**7**) [13], (3*S*,5*R*,6*S*,7*E*)-5,6-epoxy-3-hydroxy-7-megastigmen-9-one (**8**) [14], 3-hydroxy-7,8-dehydro-*β*-ionol (**9**) [15], phaseic acid (**10**) [16], loliolida (**11**) [17], ( +)-syringaresinol (**12**) [18], medioresinol (**13**) [19], (-)-pinoresinol (**14**) [20], (1R*,2R*,5R*,6S*)**-**6-(4-hydroxy-3-methoxyphenyl)-3,7-dioxabicyclo[3.3.0]octan-2-ol (**15**) [21], ( +)-lariciresinol (**16**) [22], ( +)-isolariciresinol (**17**) [23], meliasendanin B (**18**) [24], evofolin B (**19**) [25], vanillic acid (**20**) [26], 4-hydroxybenzoic acid (**21**) [27], syringic acid (**22**) [28], 4-hydroxyacetophenone (**23**) [29], ferulic acid (**24**) [30], *p*-coumaric acid (**25**) [31], caffeic acid methyl ester (**26**) [32], tyrosol (**27**) [33], ethyl 3,4-dihydroxybenzoate (**28**) [34], *β-*hydroxy propiovanillone (**29**) [35], 6,7-dimethoxy-4-hydroxy-1-naphthoic acid (**30**) [36], chrysoeriol (**31**) [37], scopoletin (**32**) [38], 5-hydroxy-4-phenyl-5*H*-furan-2-one (**33**) [39], 4,4-dimethylheptanedioic acid (**34**) [40].

### The establishment of lipopolysaccharide (LPS)-induced MH7A inflammation model.

**Fig. 4:**
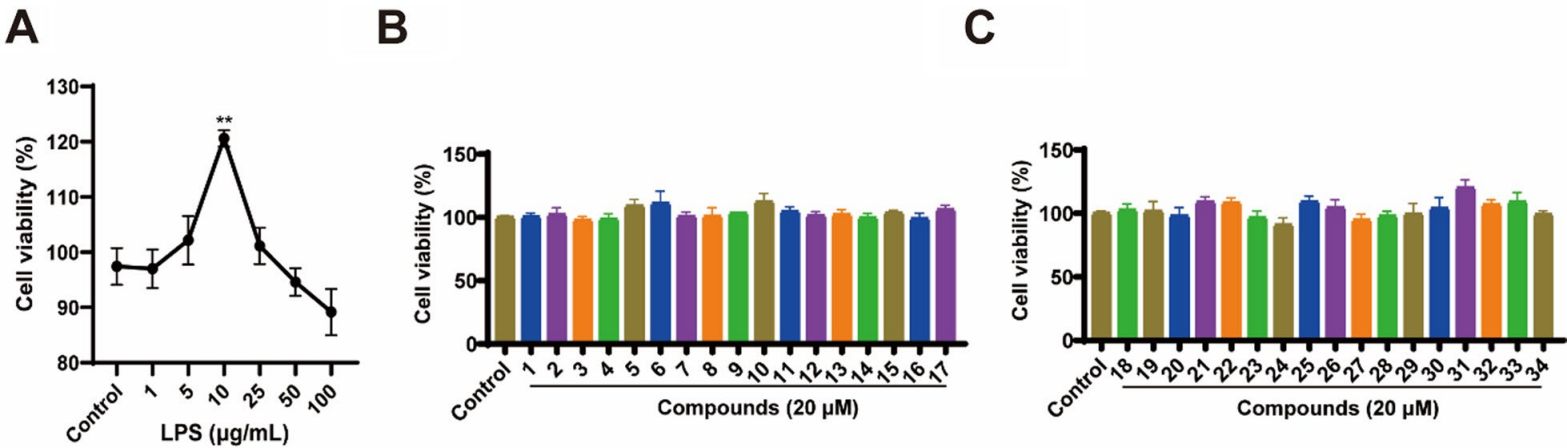
**A** Cell viability of MH7A cells under stimulation of different concentrations of LPS. **B-C** The cell viability of compounds on MH7A cells. Compared with the control group, ** *p* < 0.01

Previous studies showed that RA-FLS were stimulated by chemotactic factors, including IL-1β, TNF-α, and LPS [41, 42]. Additionally, LPS-induced RA-FLS displayed biological traits that were associated with RA, such as aberrant proliferation and resistance to cell death, which were similar to those of tumor cells [43]. Therefore, the immortalized RA-FLS MH7A cell line induced by LPS was utilized for in vitro studies to investigate the therapeutic effects of isolated compounds of *L. barbarum* leaves on RA. Following a 24-hour exposure to varying doses of LPS (1, 5, 10, 25, 50 and 100 μg/mL), the MTT test was used to assess the viability of the cells. As depicted in Fig. 4A, a peak cell viability of 120.8% was reached with an LPS concentration of 10 μg/mL, showing a significant difference compared to the control group (*p* < 0.01). As a result, for the subsequent stimulation, 10 μg/mL of LPS was selected.

### Inhibitory effect of isolated compounds on proliferation, nitric oxide (NO) lactate dehydrogenase (LDH) production in LPS-induced MH7A cells.

**Fig. 5:**
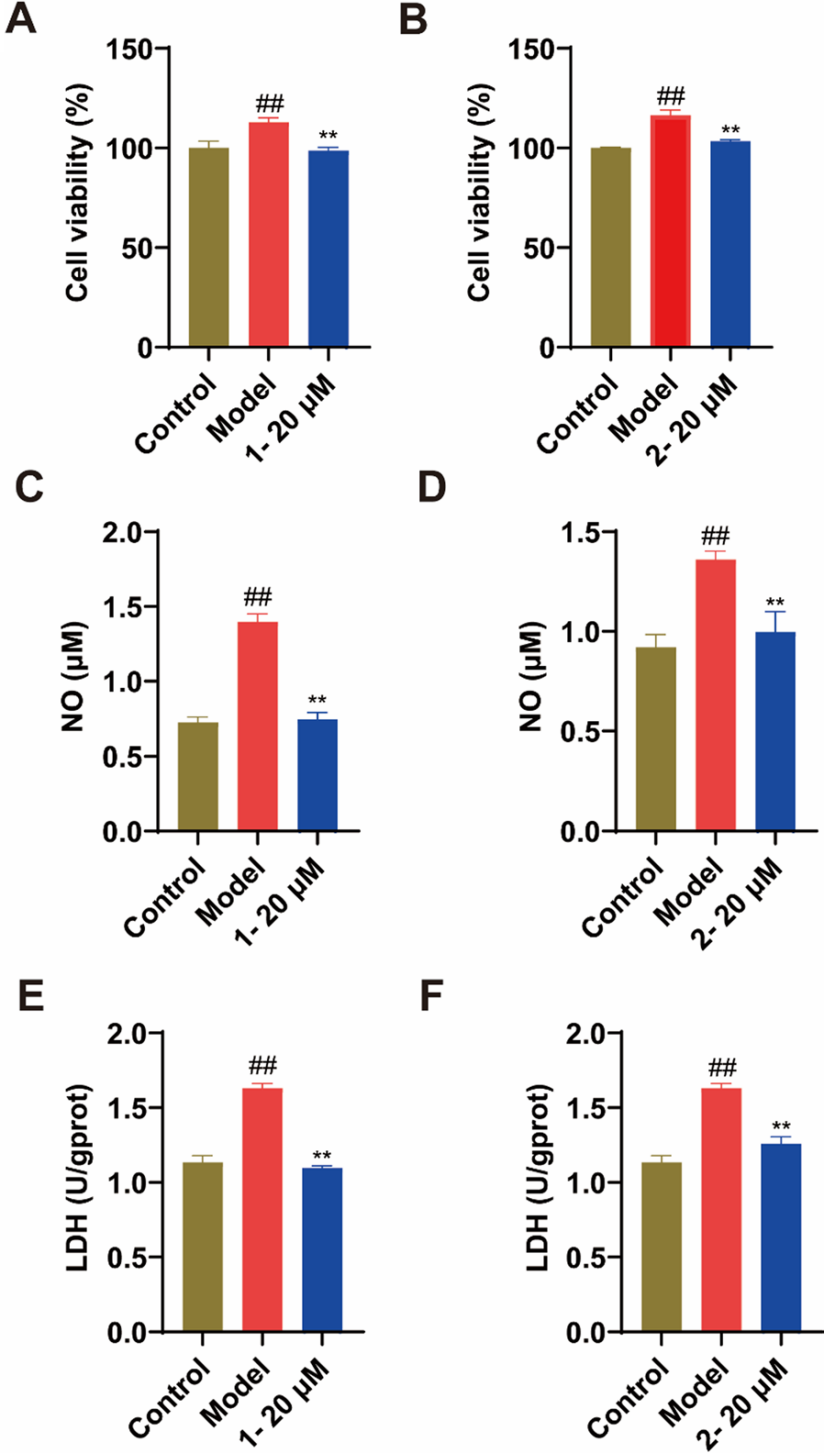
The cell viabilities of compounds **1** and **2** on MH7A by LPS induction (**A**-**B**). The effect of compounds **1** and **2** on the production of NO (**C**-**D**) and the release of LDH (**E**–**F**) in LPS-induced MH7A. Compared with the control group, ## *p* < 0.01; compared with the model group, ** *p* < 0.01, respectively

Compounds were subjected to cytotoxicity testing, revealing no inhibition at a concentration of 20 μM for all (Fig. 4B–C). Furthermore, compounds **1–4**, **6**, **8–14**, **16–21**, **23–24**, and **29–33** demonstrated substantial inhibitory effects on the proliferation bioactivity of LPS-induced MH7A cells (*p* < *0.05*/*0.01*, Table S1). It was worth mentioning that the cell viabilities of compounds **1** and **2** were 112.91% and 116.47%, respectively (Fig. 5A–B). Synovial cells were considered the primary producers of NO in RA. Synovial fibroblasts were induced by pro-inflammatory cytokines to produce NO, thereby enhancing the production of inflammation [44]. LDH, a cytoplasmatic enzyme, present in essentially all organ systems is thought to be released only after cell death and local inflammation of cells may be a potential source of elevation of LDH [45]. As cells underwent proptosis, pores formed within them, resulting in an elevation of LDH levels [46]. Stimulation with LPS on MH7A cells led to elevated levels of NO and LDH production by MH7A cells compared to the control group, whereas compounds **1–3**, **6**, **8**, **10–12**, **14, 16–19**, **21**, **29** and **31** displayed inhibitory effects on NO (*p* < *0.05*/*0.01*, Table S2), with compounds **1–3**, **6**, **8–10**, **14**, **17–19, 29** and **31**–**32** also showing inhibitory effects on LDH release (*p* < *0.05*/*0.01*, **Table S3**). The compounds **1–3**, **6**, **8**, **10**, **14**, **17–19**, **29** and **31** exhibited effectiveness on LDH and NO indicators (*p* < *0.05*/*0.01*, Table S4). Among them, compounds **1** and **2** exhibited good inhibitory activity on the two indicators, NO inhibition rates were 46.7% and 26.8% respectively, and LDH inhibition rates were 32.8% and 22.8% respectively (Fig. 5C–F).

## Experimental

### General experimental procedures

NMR, HRESIMS, UV, and IR spectra were obtained following the previously described methods [47].

### Plant material

The origin of the plant material is detailed in the **Plant Material** section of Supplementary Material.

### Extraction and purification

The detailed procedures for extraction and isolation can be found in the **Extraction and purification** section of Supplementary Material.

Lycibarin A (**1**)*.* white powder; ${[\alpha ]}_{D}^{22}$−72.9 **(**0.073, MeOH**);** UV (MeOH) *λ*_max_ (log *ε*): 292 (3.80) nm; ECD (MeOH) *λ*_max_ (Δ*ε*) 210 (+ 14.60), 245 (–8.78) nm; IR *v*_max_ 1746, 1652, 1433, 1047, 693 cm^‒1^; ^1^H NMR and ^13^C NMR data see Table 1**.** HRESIMS *m*/*z* 288.1221[M + H]^+^ (calcd. for C_16_H_18_NO_4_, 288.1230).

Lycibarin B (**2**). white power; ${[\alpha ]}_{D}^{22}$−57.2 (0.056, MeOH); UV (MeOH) *λ*_max_ (log *ε*): 288 (3.54) nm; ECD (MeOH) *λ*_max_ (Δ*ε*) 209 (-1.08), 252 (+ 0.32) nm; IR *v*_max_ 3434, 1713, 1630, 1459, 1384, 1149, 1081 cm^‒1^; ^1^H and ^13^C NMR data see Table 1; HRESIMS* m*/*z* 329.1698[M + Na]^+^(calcd. for C_18_H_26_O_4_Na, 329.1723).

### Cell culture

MH7A (RA-FLS) cell line was obtained from Jennio Biotech Co., Ltd. (Guangzhou, China) and cultured at 37℃ with 5% CO_2_ in DMEM (Gbico, USA) supplemented with 10% FBS (Procell, China), 100 U/mL penicillin, and 100 μg/mL streptomycin.

### LPS-induced MH7A cells inflammation model

The MTT test was used to assess the cell viability of LPS on MH7A cells [48]. Each well was initially seeded with 1.5 × 10^4^ cells, which were then spilt into control and LPS groups. The cells were incubated for 24 h before being treated to different doses of LPS (1, 5, 10, 25, 50 and 100 μg/mL) for another 24 h. Subsequently, 100 μL of MTT (Aladdin, Shanghai, China) was added at 0.5 mg/mL concentration and continued for 4 h. The supernatant was sucked out, and 100 μL DMSO was added to each well, followed by a 10-min incubation. The absorbance cell medium was measured at 490 nm using microplate reader (Molecular Devices, Shanghai, China).

### Cell viability assay

In short, 100 μL of MH7A cell suspension (1.5 × 10^5^ cells/mL) was added to a 96-well plate. Upon reaching 70%-80% confluence, the cells were exposed to the compounds at a 20 μM concentration for 24 h.

### Inhibition of compounds on LPS-induced MH7A cells proliferation

According to the results of viability, MH7A cells were seeded in 96-cell plates for 24 h incubation and divided into control, LPS (model group), LPS and different compounds (20 μM). All groups, except for the control, were stimulated with LPS for 24 h. The steps that followed for evaluating cell viability were in accordance with the instructions outlined in previous section [49].

### NO assay

After being planted in 96-well plates at a density of 1.5 × 10^4^ cells per cell, MH7A cells were incubated for 24 h. Following this, the cells were stimulated with LPS (10 μg/mL) and treated with the tested compounds at a concentration of 20 μM. A microplate reader (Shanghai, Molecular Devices, China) was used to quantify NO at 540 nm after 50 μL of the supernatant from each well as moved to a new 96-well plate following a 24-hour incubation period [50].

### LDH measurement

MH7A cells were seeded in 6-well plates and incubated with LPS and the tested compounds for 24 h. Removed the cell culture supernatant, collected the cells, and then performed the measurement according to the LDH assay kit [49].

## Supplementary Information

Additional file 1. The spectral data of known compounds **3-34**; 1D and 2D NMR, HRESIMS, IR, CD and UV spectra of new compounds **1**-**2**; the ECD calculation details of new compounds; inhibitory effects of 34 compounds on proliferation, NO, LDH production in LPS-induced MH7A cells.

## Data Availability

The data supporting the results of this study can be obtained from the corresponding author on reasonable request.
