# Supplementary material for: Chemical constituents of Lycium barbarum leaves and their anti-rheumatoid arthritis activity in vitro
Source: Nat Prod Bioprospect. 2025 May 30;15(1):35. doi: 10.1007/s13659-025-00516-9 (PMC12125459; doi:10.1007/s13659-025-00516-9)
Supplement: Supplementary file 1 — Additional file 1. The spectral data of known compounds 3-34; 1D and 2D NMR, HRESIMS, IR, CD and UV spectra of new compounds 1-2; the ECD calculation details of new compounds; inhibitory effects of 34 compounds on proliferation, NO, LDH production in LPS-induced MH7A cells. [file 13659_2025_516_MOESM1_ESM.docx]

**Supporting Information for**

**Chemical constituents of Lycium barbarum leaves and their anti-rheumatoid arthritis activity *in vitro***

Zi-Jiao Wang^a,c^, Bang-Yin Tan^a,c^, Yun Zhao^a,c^, Chang-Bin Wang^a,c^, Yun-Li Zhao^b,*^, and Xiao-Dong Luo^a,b,*^

*^a^* *State Key Laboratory of Phytochemistry and Natural Medicine, Kunming Institute of Botany, Chinese Academy of Sciences, Kunming 650201, P. R. China*

*^b Yunnan Characteristic Plant Extraction Laboratory Co. Ltd; Key Laboratory of Medicinal Chemistry for Natural Resource, Ministry of Education; Yunnan Key Laboratory of Research and Development for Natural Products; School of Pharmacy; School of Chemical Science and Technology, Yunnan University,^* *^Southwest United Graduate School, Kunming, 650091, P. R. China^*

*^c^ University of Chinese Academy of Sciences, Beijing 100049, P. R. China*

__________________________________________________

* Corresponding author.

Tel.: +86-0871-6522317;

*E-mail address:* [xdluo@mail.kib.ac.cn](mailto:xdluo@mail.kib.ac.cn)

**Contents**

[**The identification data for known compounds** 3](#_Toc193821174)

[**Figure S1**. ^1^H NMR spectrum (600 MHz, CDCl_3_) of **1** 11](#_Toc193821175)

[**Figure S2.** ^13^C NMR and DEPT spectrum (150 MHz, CDCl_3_) of **1** 11](#_Toc193821176)

[**Figure S3.** HSQC spectrum (600 MHz, CDCl_3_) of **1** 12](#_Toc193821177)

[**Figure S4**. HMBC spectrum (600 MHz, CDCl_3_) of **1** 12](#_Toc193821178)

[**Figure S5.** ^1^H-^1^H COSY spectrum (600 MHz, CDCl_3_) of **1** 13](#_Toc193821179)

[**Figure S6.** NOESY spectrum (600 MHz, CDCl_3_) of **1** 13](#_Toc193821180)

[**Figure S7.** IR spectrum of **1** 14](#_Toc193821181)

[**Figure S8.** HR-ESIMS spectrum of **1** 14](#_Toc193821182)

[**Figure S9.** UV spectrum of compound **1** 15](#_Toc193821183)

[**Figure S10.** Circular dichroism spectrum of **1** 15](#_Toc193821184)

[**Figure S11.** ^1^H NMR spectrum (600 MHz, CD_3_OD) of **2** 16](#_Toc193821185)

[**Figure S12.** ^13^C NMR and DEPT spectrum (150 MHz, CD_3_OD) of **2** 16](#_Toc193821186)

[**Figure S13.** HSQC spectrum (600 MHz, CD_3_OD) of **2** 17](#_Toc193821187)

[**Figure S14.** HMBC spectrum (600 MHz, CD_3_OD) of **2** 17](#_Toc193821188)

[**Figure S15.** ^1^H-^1^H COSY spectrum (600 MHz, CD_3_OD) of **2** 18](#_Toc193821189)

[**Figure S16.** NOESY spectrum (600 MHz, CD_3_OD) of **2** 18](#_Toc193821190)

[**Figure S17.** IR spectrum of **2** 19](#_Toc193821191)

[**Figure S18**. HRESIMS spectrum of **2** 19](#_Toc193821192)

[**Figure S19.** UV spectrum of compound **2** 20](#_Toc193821193)

[**Figure S20.** Circular dichroism spectrum of **2** 20](#_Toc193821194)

[**ECD Calculation Details.** 21](#_Toc193821195)

[**Plant material** 21](#_Toc193821196)

[**Extraction and purification** 21](#_Toc193821197)

[**Table S1.** The cell viabilities of 34 compounds on MH7A by LPS induction 24](#_Toc193821198)

[**Table S2.** The effect of 24 compounds on the production of NO in LPS-induced MH7A 25](#_Toc193821199)

[**Table S3.** The effect of 24 compounds on the release of LDH in LPS-induced MH7A 27](#_Toc193821200)

[**Table S4.** The summary of effective compounds on both NO and LDH 28](#_Toc193821201)

**The identification data for known compounds**

**(-)-(3*S*,4*S*)-eucomegastigmane B (3)**: C_13_H_20_O_3_; white amorphous powder; ^1^H NMR (400 MHz, DMSO-*d*_6_) *δ*_H_: 1.02 (3H, s, H-13), 1.08 (3H, s, H-12), 1.42 (1H, t, *J* = 12.2 Hz, H-2*α*), 1.60 (1H, dd, *J* = 12.8, 3.5 Hz, H-2*β*), 1.74 (3H, s, H-11), 2.27 (3H, s, H-10), 3.55 (1H, ddd, *J* = 11.2, 7.4, 3.6 Hz, H-3), 3.63 (1H, d, *J* = 7.4 Hz, H-4), 6.04 (1H, d, *J* = 16.5 Hz, H-8), 7.16 (1H, d, *J* = 16.5 Hz, H-7); ^13^C NMR (100 MHz, DMSO-*d*_6_) *δ*_C_: 17.3 (q, C-11), 27.7 (q, C-10), 28.4 (q, C-12), 30.5 (q, C-13), 36.4 (s, C-1), 45.7 (t, C-2), 69.4 (d, C-3), 76.6 (d, C-4), 132.8 (d, C-8), 136.2 (s, C-5), 136.8 (s, C-6), 142.5 (d, C-7), 198.4 (s, C-9); positive ESI-MS *m*/*z* 247 [M+Na]^+^.

***cis*-3,6-dihydroxy-*α*-ionone (4)**: C_13_H_20_O_3_; colorless oil; ^1^H NMR (600 MHz, DMSO-*d*_6_) *δ*_H_: 0.84 (3H, s, H-11), 0.85 (3H, s, H-12), 1.54 (3H, s, H-13), 1.57 (1H, dd, *J* = 13.1, 5.4 Hz, H-2*α*), 1.63 (1H, dd, *J* = 13.1, 7.2 Hz, H-2*β*), 2.22 (3H, s, H-10), 4.76 (1H, br s, H-3), 5.49 (1H, s, H-4), 6.22 (1H, d, *J* = 16.4 Hz, H-8), 6.72 (1H, d, *J* = 16.4 Hz, H-7); ^13^C NMR (150 MHz, DMSO-*d*_6_) *δ*_C_: 19.0 (q, C-13), 24.4 (q, C-12), 24.6 (q, C-11), 27.2 (q, C-10), 37.7 (s, C-1), 63.1 (d, C-3), 76.2 (s, C-6), 128.8 (d, C-4), 129.9 (d, C-8), 135.1 (d, C-5), 150.4 (s, C-7), 198.6 (s, C-9); positive ESI-MS *m*/*z* 247 [M+Na]^+^.

**(+)-dehydrovomifoliol (5):** C_13_H_18_O_3_; yellow oil; ^1^H NMR (400 MHz, DMSO-*d*_6_) *δ*_H_: 0.92 (3H, s, H-12), 0.96 (3H, s, H-11), 1.81 (3H, s, H-13), 2.17 (1H, d, *J* =17.6 Hz, H-2*β*), 2.27 (3H, s, H-10), 2.64 (1H, d, *J* =17.6 Hz, H-2*α*), 5.86 (1H, s, H-4), 6.28 (1H, d, *J* = 16.0 Hz, H-8), 6.98 (1H, d, *J* = 16.0 Hz, H-7); ^13^C NMR (100 MHz, DMSO-*d*_6_) *δ*_C_: 19.0 (q, C-13), 23.6 (q, C-12), 24.6 (q, C-11), 27.8 (q, C-10), 49.6 (t, C-2), 78.6 (s, C-6), 127.0 (d, C-4), 131.0 (d, C-8), 147.6 (d, C-7), 162.1 (s, C-5), 197.5 (s, C-3), 198.7 (s, C-9); positive ESI-MS *m*/*z* 245 [M+Na]^+^.

**vomifoliol (6)**: C_13_H_20_O_3_; colorless needles; ^1^H NMR (400 MHz, DMSO-*d*_6_) *δ*_H_: 1.01 (3H, s, H-11), 1.08 (3H, s, H-12), 1.30 (3H, d, *J* = 6.8 Hz, H-10), 1.89 (3H, s, H-13), 2.23 (1H, d, *J* = 17.2 Hz, H-2*α*), 2.45 (1H, d, *J* = 17.2 Hz, H-2*β*), 4.41 (1H, m, H-9), 5.81 (2H, m, H-7, 8), 5.90 (1H, s, H-4); ^13^C NMR (100 MHz, DMSO-*d*_6_) *δ*_C_: 19.0 (q, C-13), 23.9 (q, C-12), 24.1 (q, C-11), 24.1 (q, C-10), 40.9 (s, C-1), 49.4 (t, C-2), 66.1 (d, C-9), 77.8 (s, C-6), 125.5 (d, C-4), 127.8 (d, C-8), 135.8 (d, C-7), 164.4 (s, C-5), 197.4 (s, C-3); positive ESI-MS *m*/*z* 247 [M+Na]^+^.

***trans, trans*-abscisic acid (7)**: C_15_H_20_O_4_; colorless oil; ^1^H NMR (400 MHz, DMSO-*d*_6_) *δ*_H_: 0.90 (3H, s, H-13), 0.95 (3H, s, H-12), 1.80 (3H, s, H-14), 2.12 (1H, d, *J* = 17.1 Hz, H-2*α*), 2.19 (3H, br s, H-15), 2.55 (1H, d, *J* = 17.1 Hz, H-2*β*), 5.80 (2H, s, H-4, 10), 6.20 (1H, d, *J* = 15.6 Hz, H-7), 6.40 (1H, d, *J* = 15.6 Hz, H-8); ^13^C NMR (100 MHz, DMSO-*d*_6_) *δ*_C_: 13.7 (q, C-15), 18.8 (q, C-14), 23.2 (q, C-12), 24.2 (q, C-13), 41.3 (s, C-1), 49.4 (t, C-2), 78.3 (s, C-6), 120.0 (d, C-10), 125.9 (d, C-4), 133.3 (d, C-8), 135.2 (d, C-7), 149.6 (s, C-5), 163.1 (s, C-9), 168.5 (s, C-11), 197.3 (s, C-3); positive ESI-MS *m*/*z* 287 [M+Na]^+^.

**(3*S*,5*R*,6*S*,7*E*)-5,6-epoxy-3-hydroxy-7-megastigmen-9-one (8)**: C_13_H_20_O_3_; red oil; ^1^H NMR (400 MHz, DMSO-*d*_6_) *δ*_H_: 0.86 (3H, s, H-12), 1.11 (3H, s, H-11), 1.12 (3H, s, H-13), 1.19 (1H, m, H-2*α*), 1.46 (1H, m, H-2*β*), 1.57 (1H, m, H-4*β*), 2.19 (1H, dd, *J* = 14.4, 4.8 Hz, H-4*α*), 2.24 (3H, s, H-10), 3.60 (1H, m, H-3), 6.01 (1H, d, *J* = 15.6 Hz, H-7), 7.07 (1H, d, *J* = 15.6 Hz, H-8); ^13^C NMR (100 MHz, DMSO-*d*_6_) *δ*_C_: 20.1 (q, C-13), 25.2 (q, C-12), 27.6 (q, C-11), 29.5 (q, C-10), 35.0 (s, C-1), 40.8 (t, C-2), 47.0 (t, C-4), 63.2 (d, C-3), 67.4 (s, C-5), 69.4 (s, C-6), 132.7 (d, C-8), 143.8 (d, C-7), 197.8 (s, C-9); positive ESI-MS *m*/*z* 247 [M+Na]^+^.

**3-hydroxy-7,8-dehydro-*β*-ionol (9)**: C_13_H_20_O_2_; white amorphous powder; ^1^H NMR (400 MHz, DMSO-*d*_6_) *δ*_H_: 1.04 (3H, s, H-11), 1.08 (3H, s, H-12), 1.25 (1H, t, *J* = 12.0 Hz, H-2*β*), 1.33 (3H, d, *J* = 6.5 Hz, H-10), 1.69 (1H, m, H-2*α*), 1.80 (3H, s, H-13), 1.89 (1H, dd, *J* = 17.6, 9.6 Hz, H-4*β*), 2.24 (1H, dd, *J* = 17.5, 4.9 Hz, H-4*α*), 3.72 (1H, m, H-3), 4.52 (1H, q, *J* = 6.0 Hz, H-9); ^13^C NMR (100 MHz, DMSO-*d*_6_) *δ*_C_: 22.6 (q, C-13), 25.6 (q, C-10), 28.9 (q, C-11), 30.8 (q, C-12), 36.4 (s, C-1), 41.6 (t, C-4), 47.0 (t, C-2), 57.2 (d, C-9), 63.0 (d, C-3), 80.8 (s, C-8), 97.4 (s, C-7), 123.3 (s, C-6), 137.7 (s, C-5); positive ESI-MS *m*/*z* 209 [M+H]^+^.

**phaseic acid (10):** C_15_H_20_O_5_; colorless crystals; ^1^H NMR (400 MHz, DMSO-*d*_6_) *δ*_H_: 0.88 (3H, s, H-9'), 1.08 (3H, s, H-7'), 2.23-2.35 (2H, m, H-5), 2.69 (1H, dd, *J* = 18.0, 2.0 Hz, H-3'*α*),2.79 (1H, d, *J* = 18.0 Hz, H-3'*β*), 3.52 (1H, d, *J* = 7.6 Hz, H-8'*α*), 3.78 (1H, dd, *J* = 7.6, 2.8 Hz, H-8'*β*), 5.69 (1H, s, H-2), 6.44 (1H, d, *J* = 16.0 Hz, H-5), 7.98 (1H, d, *J* = 16.0 Hz, H-4); ^13^C NMR (100 MHz, DMSO-*d*_6_) *δ*_C_: 15.8 (q, C-9'), 19.6 (q, C-7'), 21.3 (q, C-6), 48.7 (s, C-6'), 52.4 (t, C-5'), 53.5 (t, C-3'), 77.1 (t, C-8'), 86.5 (s, C-2'), 119.2 (d, C-2), 131.1 (d, C-4), 134.0 (d, C-5), 149.6 (d, C-3), 167.5 (s, C-1); positive ESI-MS *m*/*z* 281 [M+H]^+^.

**loliolida (11):** C_11_H_16_O_3;_ amorphous powder (MeOH); ^1^H NMR (400 MHz, DMSO-*d*_6_) *δ*_H_: 1.19 (3H, s, H-10), 1.37 (3H, s, H-9), 1.41 (1H, dd, *J* = 14, 3.6 Hz, H-2*α*), 1.63 (1H, dd, *J* = 13.6, 4 Hz, H-4*α*), 1.66 (3H, s, H-11), 1.86 (1H, dt, *J* = 14, 2.4 Hz, H-2*β*), 2.29 (1H, dt, *J* = 13.6, 2 Hz, H-4*β*), 4.08 (1H, m, H-3), 5.78 (1H, s, H-7); ^13^C NMR (100 MHz, DMSO-*d*_6_) *δ*_C_: 26.7 (q, C-9), 27.3 (q, C-11), 30.9 (q, C-10), 36.1 (s, C-1), 45.7 (t, C-4), 47.1 (t, C-2), 65.3 (d, C-3), 86.8 (s, C-5), 112.6 (d, C-7), 171.5 (s, C-8), 183.5 (s, C-6); positive ESI-MS *m*/*z* 197 [M+H]^+^.

**(+)-syringaresinol (12):** colorless needles, C_22_H_26_O_8_, ^1^H NMR (400 MHz, DMSO-*d*_6_) *δ*_H_: 3.05 (2H, m, H-9*β*, 9'*β* ), 3.77 (12H, s, 3, 3', 5, 5'-OCH_3_), 3.78 (2H, overlapped, H-9α, 9'α), 4.61 (2H, d, *J* = 3.6 Hz, H-7, 7'), 6.60 (4H, s, H-2, 2', 6, 6'), 8.30 (2H, s, 4, 4'-OH); ^13^C NMR (100 MHz, DMSO-*d*_6_) *δ*_C_: 54.1 (d, C-8, 8'), 56.5 (q, 3', 3', 5, 5'-OCH_3_ ), 71.5 (d, C-9, 9'), 85.8 (d, C-7, 7'), 104.1 (d, C-2', 2', 6, 6'), 131.9 (s, C-1, 1'), 135.3 (s, C-4, 4'), 148.4 (s, C-3, 3', 5, 5'); positive ESI-MS *m*/*z* 419 [M+H]^+^.

**medioresinol (13):** white amorphous powder; C_21_H_24_O_7_, ^1^H NMR (400 MHz, DMSO-*d*_6_) *δ*_H_: 3.05 (2H, s, H-8, 8'), 3.75 (2H, m, H-9*β*, H-9'*β*), 3.76 (9H, s, 3-OCH_3_, 3'-OCH_3_, 5'-OCH_3_), 4.14 (2H, m, H-9α, 9'α), 4.61 (2H, br s, H-7, 7'), 6.60 (2H, s, H-2', 6'), 6.72 (1H, m, H-5), 6.76 (1H, m, H-6), 6.89 (1H, d, *J* = 1.2 Hz, H-2), 8.70 (1H, s, 4-OH), 8.80 (1H, s, 4'-OH); ^13^C NMR (100 MHz, DMSO-*d*_6_) *δ*_C_: 53.5 (d, C-8'), 54.1 (d, C-8), 56.1 (q, 3-OCH_3_), 56.5 (q, 3'-OCH_3_, 5'-OCH_3_), 71.4 (t, C-9'), 71.5 (t, C-9), 85.6 (d, C-7), 85.8 (d, C-7), 104.1 (d, C-2', C-6'), 110.9 (d, C-2), 115.6 (d, C-5), 119.1 (d, C-6), 131.9 (s, C-1'), 132.7 (s, C-1), 135.3 (s, C-4'), 145.9 (s, C-4), 146.4 (s, C-3), 147.8 (s, C-3'), 147.9 (s, C-3); positive ESI-MS *m*/*z* 389 [M+H]^+^.

**(-)-pinoresinol (14):** C_20_H_22_O_6_, yellow amorphous powder, ^1^H NMR (400 MHz, DMSO-*d*_6_) *δ*_H_: 3.03 (2H, m, H-8, 8'), 3.75 (2H, m, H-9*β*, H-9'*β*), 3.76 (6H, s, 3-OCH_3_, 3'-OCH_3_), 4.12 (2H, m, H-9*α*, 9'α), 4.61 (2H, d, *J* = 4.1 Hz, H-7, 7'), 6.72 (2H, m, H-6, 6'), 6.89 (2H, br s, H-2, 2'), 8.9 (2H, br s, 4', 4''-OH); ^13^C NMR (100 MHz, DMSO-*d*_6_) *δ*_C_: 54.1 (d, C-8'), 56.1 (q, 3, 3'-OCH_3_), 71.4 (t, C-9, 9'), 85.6 (d, C-7, 7'), 110.9 (d, C-5, 5'), 115.6 (d, C-2, 2′), 119.1 (d, C-6, 6'), 132.7 (s, C-1, 1'), 146.4 (s, C-4, C-4'), 148.0 (s, C-3, 3'); positive ESI-MS *m*/*z* 359 [M+H]^+^.

**(1*R**,2*R**,5*R**,6*S**)-6-(4-hydroxy-3-methoxyphenyl)-3,7-dioxabicyclo[3.3.0] octan-2-ol** (**15**): C_13_H_16_O_5_; white amorphous powder; ^1^H NMR (400 MHz, DMSO-*d*_6_) *δ*_H_: 2.86 (1H, m, H-7), 3.06 (1H, m, H-3), 3.40 (1H, m, H-4*β*), 3.77 (1H, m, H-8*β*), 3.83 (3H, s, 5'-OCH_3_), 3.95 (1H, dd, *J* = 8.8, 6.0 Hz, H-4*α*), 4.23 (2H, m, H-6, 8*α*), 5.19 (1H, d, *J* = 3.8 Hz, H-2), 6.72 (2H, m, H-3', 6′), 6.86 (1H, m, H-2'); ^13^C NMR (150 MHz, DMSO-*d*_6_) *δ*_C_: 52.3 (d, C-3), 53.5 (d, C-7), 55.5 (q, 5'-OCH_3_), 68.0 (t, C-4), 70.8 (t, C-8), 86.3 (d, C-6), 101.1 (d, C-2), 110.2 (d, C-6'), 115.1 (d, C-2'), 118.7 (d, C-3'), 132.1 (s, C-1'), 146.0 (s, C-4'), 147.5 (s, C-5'); positive ESI-MS *m*/*z* 253 [M+H]^+^.

**(+)-lariciresinol (16):** white amorphous powder; C_20_H_24_O_6_, ^1^H NMR (400 MHz, DMSO-*d*_6_) *δ*_H_: 2.17 (1H, m, H-8'), 2.40 (1H, m, H-7*α*), 2.57 (1H, m, H-8), 2.82 (1H, dd, *J* = 13.2, 4.8 Hz, H-7*β*) , 3.45 (1H, m, H-9'*β*), 3.55 (1H, dd, *J* = 8.0, 6.8 Hz, H-9*α*), 3.65 (1H, m, H-9'*α*), 3.74 (3H, s, 3'-OCH_3_), 3.74 (3H, s, 3-OCH_3_), 3.86 (1H, dd, *J* = 8.0, 6.4 Hz, H-9*β*), 4.64 (1H, d, *J* = 6.4 Hz, H-7'), 4.68 (1H, overlapped, 9'-OH), 6.56 (1H, dd, *J* = 8.0, 1.6 Hz, H-6), 6.67 (2H, m, H-5, 5'), 6.69 (1H, br s, H-6'), 6.74 (1H, d, *J* = 0.9 Hz, H-2), 6.82 (1H, d, *J* = 1.7 Hz, H-2'), 8.70 (1H, s, 4-OH), 8.80 (1H, s, 4'-OH); ^13^C NMR (100 MHz, DMSO-*d*_6_) *δ*_C_: 32.7 (d, C-7), 42.4 (d, C-8), 52.4 (d, C-8'), 56.0 (q, 3'-OCH_3_), 56.0 (q, 3-OCH_3_), 59.0 (t, C-9'), 72.3 (t, C-9), 82.2 (d, C-7'), 110.4( d, C-2), 113.2 (d, C-2), 115.5 (d, C-5), 115.8 (C-5'), 118.6 (d, C-6'), 121.0 (d, C-6), 132.2 (s, C-1), 135.2 (s, C-1'), 145.0 (s, C-4), 146.0 (s, C-4'), 147.8 (s, C-3), 147.9 (s, C-3'); positive ESI-MS *m*/*z* 361 [M+H]^+^.

**(+)-isolariciresinol (17):** C_20_H_24_O_6_; white amorphous powder; ^1^H NMR (400 MHz, DMSO-*d*_6_) *δ*_H_: 1.61 (1H, m, H-8), 1.83 (1H, m, H-8'), 2.67 (2H, m, H-7'), 3.16 (1H, m, H-9*β*), 3.43 (1H, m, H-9'*β*), 3.45 (1H, m, H-9*α*), 3.56 (1H, m, H-9'*α*), 3.69 (3H, s, 4'-OCH_3_), 3.70 (3H, s, 3-OCH_3_), 3.74 (1H, d, *J* = 10.4 Hz, H-7), 6.08 (1H, s, H-5'), 6.48 (1H, dd, *J* = 8.4, 1.6 Hz, H-5), 6.63 (1H, d, *J* = 1.6 Hz, H-2), 6.68 (1H, d, *J* = 8.1 Hz, H-6); ^13^C NMR (100 MHz, DMSO-*d*_6_) *δ*_C_: 32.7 (t, C-7'), 38.5 (d, C-8'), 46.4 (d, C-7, 8), 56.0 (q, 4'-OCH_3_), 56.1 (q, 3-OCH_3_), 60.2 (t, C-9), 64.0 (t, C-9'), 112.5 (d, C-2'), 113.7 (d, C-2), 115.7 (d, C-5), 116.7 (d, C-5') 121.9 (d, C-6), 127.6 (s, C-1'), 137.6 (s, C-1), 144.6 (s, C-4'), 145.1 (s, C-4), 146.0 (s, C-3'), 147.8 (s, C-3); positive ESI-MS *m*/*z* 383 [M+Na]^+^.

**meliasendanin B (18):** C_20_H_24_O_8_; white amorphous powder; ^1^H NMR (400 MHz, DMSO-*d*_6_) *δ*_H_: 3.22 (1H, m, H-8), 3.36 (1H, m, H-9*β*), 3.44 (1H, m, H-9*α*), 3.48 (1H, m, H-8'), 3.61 (1H, m, H-9'*β*), 3.69 (1H, m, H-9'*α*), 3.75 (3H, s, 3-OCH_3_), 3.77 (3H, s, 3'-OCH_3_), 4.44 (2H, brs, H-9), 4.51 (1H, s, H-8), 4.97 (1H, m, H-8'), 5.02 (1H, m, H-7), 5.43 (1H, d, *J* = 6.8 Hz, H-7'), 6.76 (2H, br s, H-5', 6'), 6.83 (1H, s, H-6), 6.86 (1H, s, H-2), 6.92 (1H, s, H-2'), 9.01 (1H, s, 4'-OH); ^13^C NMR (100 MHz, DMSO-*d*_6_) *δ*_C_: 53.4 (d, C-8'), 56.0 (q, 3-OCH_3_), 56.1 (q, 3'-OCH_3_), 63.1 (t, C-9'), 63.6 (t, C-9), 73.4 (d, C-7), 76.3 (d, C-8), 87.0 (d, C-7'), 110.3 (d, C-2'), 111.4 (d, C-2), 115.6 (d, C-5'), 115.7 (d, C-6), 118.9 (d, C-6'), 128.9 (s, C-5), 133.0 (s, C-1), 137.0 (s, C-1'), 143.5 (s, C-3), 146.7 (s, C-4'), 146.8 (s, C-3'), 148.0 (s, C-4); positive ESI-MS *m*/*z* 415 [M+Na]^+^.

**evofolin B (19):** C_17_H_18_O_6_; yellow oil; ^1^H NMR (400 MHz, DMSO-*d*_6_) *δ*_H_: 3.49 (1H, m, H-9*β*), 3.71 (3H, s, 3'-OCH_3_), 3.78 (3H, s, 3-OCH_3_), 4.09 (1H, m, H-9*α*), 4.73 (1H, dd, *J* = 7.6, 4.0 Hz, H-8), 6.65 (2H, m, H-5', H-6'), 6.80 (1H, d, *J* = 8.4 Hz, H-5), 6.90 (1H, d, *J* = 1.8 Hz, H-2'), 7.47 (1H, d, *J* = 2.0 Hz, H-2), 7.58 (1H, dd, *J* = 8.3, 2.0 Hz, H-6); ^13^C NMR (100 MHz, DMSO-*d*_6_) *δ*_C_: 53.9 (d, C-8), 55.5 (s, 3'-OCH_3_), 55.6 (s, 3-OCH_3_), 111.6 (d, C-2), 112.4 (d, C-2'), 114.9 (d, C-5), 115.8 (d, C-5'), 120.5 (d, C-6'), 123.6 (d, C-6), 128.3 (s, C-1'), 128.6 (s, C-1), 145.6 (s, C-4'), 147.5 (s, C-3), 147.6 (s, C-3'), 151.6 (s, C-4), 197.1 (s, C-7); positive ESI-MS *m*/*z* 341 [M+Na]^+^.

**vanillic acid (20):** C_8_H_8_O_4_; white powder; ^1^H NMR (400 MHz, DMSO-*d*_6_) *δ*_H_: 3.81 (3H, s, 3-OCH_3_), 6.85 (1H, d, *J* = 8.0 Hz, H-5), 7.45 (1H, s, H-2), 7.47 (2H, m, H-6); ^13^C NMR (100 MHz, DMSO-*d*_6_), *δ*_C_: 56.0 (q, 3-OCH_3_), 113.2 (d, C-5), 115.5 (d, C-2), 122.1 (s, C-1), 147.7 (s, C-3), 151.6 (s, C-4), 167.7 (s, COOH); positive ESI-MS *m*/*z* 169 [M+H]^+^.

**4-hydroxybenzoic acid (21):** C_7_H_6_O_3_; white amorphous powder; ^1^H NMR (400 MHz, MSO-*d*_6_) *δ*_H_: 6.83 (2H, d, *J* = 8.7 Hz, H-3, 5), 7.81 (1H, d, *J* = 8.7 Hz, H-2, 6); ^13^C NMR (100 MHz, DMSO-*d*_6_) *δ*_C_: 115.6 (d, C-3, 5), 121.8 (s, C-1), 132.0 (d, C-2, 6), 162.0 (s, C-4), 167.8 (s, COOH); positive ESI-MS *m*/*z* 139 [M+H]^+^.

**syringic acid (22)**: C_9_H_10_O_5_; colorless needles; ^1^H NMR (400 MHz, DMSO-*d*_6_), *δ*_H_: 3.83 (6H, s, 3, 5-OCH_3_), 7.30 (2H, s, H-2, 6), 9.16 (1H, br s, 4-OH), 12.51 (1H, br s, COOH); ^13^C NMR (100 MHz, DMSO-*d*_6_) *δ*_C_: 56.0 (q, 3, 5-OCH_3_), 106.8 (d, C-2, 6), 120.4 (s, C-1), 140.2 (s, C-4), 147.4 (s, C-3, 5), 167.3 (s, COOH); positive ESI-MS *m*/*z* 199 [M+H]^+^.

**4-hydroxyacetophenone (23)**: C_8_H_8_O_2_；yellow powder；^1^H NMR (400 MHz, DMSO-*d*_6_) *δ*_H_: 2.45 (3H, s, H-8), 6.86 (2H, d, *J* = 8.4 Hz, H-3, 5), 7.82 (1H, d, *J* = 8.2 Hz, H-2, 6); ^13^C NMR (100MHz, DMSO-*d*_6_) *δ*_C_: 26.5 (q, C-8), 115.2 (d, C-3, 5), 129.0 (s, C-1), 131.1 (d, C-2, 6 ), 162.5 (s, C-4), 196.5 (s, C-7); positive ESI-MS *m*/*z* 137 [M+H]^+^.

**ferulic acid (24)**: C_10_H_10_O_4_; colorless needles; ^1^H NMR (400 MHz, DMSO-*d*_6_) *δ*_H_: 3.81 (3H, s, 3-OCH_3_), 6.36 (1H, d, *J* = 15.6 Hz, H-8), 6.79 (1H, d, *J* = 8.0 Hz, H-5), 7.08 (1H, d, *J* = 8.0 Hz, H-6), 7.28 (1H, br s, H-2), 7.49 (1H, d, *J* = 16.0 Hz, H-7); ^13^C NMR (100 MHz, DMSO-*d*_6_) *δ*_C_: 56.1 (q, 3-OCH_3_), 111.6 (d, C-2), 116.0 (d, C-8), 116.1 (d, C-5), 123.3 (d, C-6), 126.2 (d, C-5), 145.0 (d, C-7), 148.3 (s, C-3), 149.5 (s, C-4), 168.5 (s, COOH); positive ESI-MS *m*/*z* 217 [M+Na]^+^.

***p*-coumaric acid (25):** C_9_H_8_O_3_; colorless needles; ^1^H NMR (400 MHz, DMSO-*d*_6_) *δ*_H_: 6.29 (1H, d, *J* = 15.9 Hz, H-8), 6.79 (2H, d, *J* = 8.4 Hz, H-3, 5), 7.49 (1H, d, *J* = 15.9 Hz, H-7), 7.51 (2H, d, *J* = 8.4 Hz, H-2, 6); ^13^C NMR (100 MHz, DMSO-*d*_6_) *δ*_C_: 115.8 (d, C-3, 5), 116.0 (d, C-8), 125.7 (s, C-1), 130.7 (d, C-2, 6), 144.6 (d, C-7), 160.1 (s, C-4), 168.4 (s, COOH); positive ESI-MS *m*/*z* 165 [M+H]^+^.

**caffeic acid methyl ester (26)**: C_10_H_10_O_4_; yellow amorphous powder; ^1^H NMR (400 MHz, DMSO-*d*_6_) *δ*_H_: 3.68 (3H, s, H-10), 6.27 (1H, d, *J* = 15.6 Hz, H-8), 6.79 (1H, d, *J* = 8.0 Hz, H-5), 7.00 (1H, d, *J* = 8.0, 1.6 Hz, H-6), 7.06 (1H, br s, H-2), 7.49 (1H, d, *J* = 15.6 Hz, H-7), 9.36 (2H, br s, 3, 4-OH); ^13^C NMR (100 MHz, DMSO-*d*_6_) *δ*_C_: 51.7 (q, C-10), 114.1 (d, C-8), 115.2 (d, C-2), 116.1 (d, C-5), 121.8 (d, C-6), 125.9 (s, C-1), 145.6 (d, C-7), 146.0 (s, C-3), 148.9 (s, C-4), 167.5 (s, C-9); positive ESI-MS *m*/*z* 217 [M+Na]^+^.

**tyrosol (27):** C_8_H_10_O_2_; brown oil; ^1^H NMR (400 MHz, DMSO- *d*_6_) *δ*_H_: 2.59 (2H, t, *J* =7.6 Hz, H-7), 3.51 (2H, t, *J* = 7.2 Hz, H-8), 6.65 (2H, m, H-2, 6), 6.98 (2H, m, H-3, 5); ^13^C NMR (100 MHz, DMSO-*d*_6_) *δ*_C_: 38.3 (t, C-7), 63.1 (t, C-8), 115.4 (d, C-3,5), 130.1 (d, C-2,6), 130.1 (s, C-4), 155.1 (s, C-1); positive ESI-MS *m*/*z* 139 [M+H]^+^.

**ethyl 3,4-dihydroxybenzoate (28):** C_9_H_10_O_4_; white amorphous powder; ^1^H NMR (400 MHz, DMSO-*d*_6_) *δ*_H_: 1.28 (3H, t, *J* = 7.2 Hz, H-9), 4.22 (2H, q, *J* = 7.2 Hz, H-8), 6.78 (1H, br s, H-5), 7.36 (1H, br s, H-6), 7.31 (1H, br s, H-2); ^13^C NMR (100 MHz, DMSO-*d*_6_) *δ*_C_: 14.3 (t, C-9), 60.0 (q, C-8), 115.2 (d, C-5), 120.7 (s, C-1), 121.7 (d, C-6), 145.0 (s, C-3), 150.3 (s, C-4), 165.6 (s, C-7); positive ESI-MS *m*/*z* 183 [M+H]^+^.

***β*-hydroxy propiovanillone (29):** C_10_H_12_O_4_; yellow oil; ^1^H NMR (400 MHz, DMSO-*d*_6_) *δ*_H_: 3.04 (2H, t, *J* = 6.4 Hz, H-2), 3.75 (2H, t, *J* = 6.4 Hz, H-3), 3.82 (3H, s, 3'-OCH_3_), 6.86 (1H, d, *J* = 8.0 Hz, H-5'), 7.43 (1H, br s, H-2'), 7.51 (1H, dd, *J* = 8.0, 1.2 Hz, H-6'); ^13^C NMR (100 MHz, DMSO-*d*_6_) *δ*_C_: 41.3 (t, C-2), 56.0 (q, 3-OCH_3_), 57.3 (t, C-3), 111.5 (d, C-2), 115.4 (d, C-5'), 123.6 (d, C-6'), 129.0 (s, C-1'), 148.0 (s, C-3'), 151.8 (s, C-4'), 197.4 (s, C-1); positive ESI-MS *m*/*z* 197 [M+H]^+^.

**6,7-dimethoxy-4-hydroxy-1-naphthoic acid (30):** C_13_H_12_O_5_; white needles, ^1^H NMR (400 MHz, DMSO-*d*_6_) *δ*_H_: 3.81 (3H, s, 7-OCH_3_), 3.81 (3H, s, 6-OCH_3_), 6.84 (1H, d, *J* = 8.4 Hz, H-3), 7.20 (1H, s, H-8), 7.43 (1H, br s, H-2), 7.44 (1H, s, H-5), 9.20 (1H, br s, 4-OH), 12.53 (1H, br s, COOH); ^13^C NMR (100 MHz, DMSO-*d*_6_) *δ*_C_: 56.0 (q, 7-OCH_3_), 56.4 (q, 6-OCH_3_), 107.3 (d, C-5), 113.3 (d, C-8), 115.5 (s, C-3), 120.8 (s, C-8*α*), 122.1 (s, C-4*α*), 123.9 (d, C-2), 140.6 (s, C-1), 147.7 (s, C-7), 147.9 (s, C-6), 151.5 (s, C-4), 167.6 (s, COOH); positive ESI-MS *m*/*z* 271 [M+Na]^+^.

**chrysoeriol (31):** C_16_H_12_O_6_; yellow solid; ^1^H NMR (400 MHz, DMSO-*d*_6_) *δ*_H_: 3.89 (3H, s, 3'-OCH_3_), 6.19 (1H, d, *J* =1.6 Hz, H-6), 6.51 (1H, d, *J* = 2.0 Hz, H-8), 6.90 (1H, s, H-3), 6.93 (1H, d, *J* = 8.8 Hz, H-5'), 7.55 (1H, s, H-2'), 7.57 (1H, d, *J* = 9.2 Hz, H-6'), 9.96 (1H, s, 4'-OH), 10.82 (1H, s, 7-OH), 12.97 (1H, s, 5-OH); ^13^C NMR (100 MHz, DMSO-*d*_6_) *δ*_C_: 56.4 (q, 3'-OCH_3_), 94.5 (d, C-8), 99.3 (d, C-6), 103.7 (d, C-3), 104.1 (s, C-10), 110.7 (d, C-2'), 116.2 (d, C-5'), 120.8 (d, C-6'), 122.0 (s, C-1'), 148.6 (s, C-3'), 151.2 (s, C-4'), 157.8 (s, C-9), 161.9 (s, C-5), 164.1 (s, C-2), 164.6 (s, C-7), 182.3 (s, C-4); positive ESI-MS *m*/*z* 323 [M+Na]^+^.

**scopoletin (32):** C_10_H_8_O_4_; yellow needles; ^1^H NMR (400 MHz, DMSO-*d*_6_) *δ*_H_: 3.81 (3H, s, 6-OCH_3_), 6.20 (1H, d, *J* = 9.6 Hz, H-3), 6.77 (1H, s, H-8), 7.20 (1H, s, H-5), 7.87 (1H, d, *J* = 9.6 Hz, H-4); ^13^C NMR (100 MHz, DMSO-*d*_6_) *δ*_C_: 56.4 (q, 6-OCH_3_), 103.2 (d, C-8), 109.9 (d, C-5), 112.1 (d, C-3), 144.8 (d, C-4), 145.6 (s, C-6), 150.0 (s, C-9), 151.5 (s, C-7), 161.1 (s, C-2); positive ESI-MS *m*/*z* 215 [M+Na]^+^.

**5-hydroxy-4-phenyl-5*H*-furan-2-one (33):** C_10_H_8_O_3_; colorless oil; ^1^H NMR (400 MHz, DMSO-*d*_6_) *δ*_H_: 7.80 (2H, m, H-2', 6'), 7.51 (3H, m, H-3', 4', 5'), 6.77 (1H, s, H-4), 6.63 (1H, s, H-2); ^13^C NMR (100 MHz, DMSO-*d*_6_) *δ*_C_: 98.4 (d, C-4), 115.3 (d, C-2), 128.5 (d, C-2', 6'), 129.1 (C-1'), 129.3 (d, C-3', 5'), 131.7 (d, C-4'), 163.4 (s, C-3), 172.2 (s, C-1); positive ESI-MS *m*/*z* 199 [M+Na]^+^.

**4,4-dimethylheptanedioic acid (34):** C_9_H_16_O_4_; white needles; ^1^H NMR (400 MHz, DMSO-*d*_6_) *δ*_H_: 1.25 (6H, br s, H-8, 9), 1.48 (4H, t, *J* = 7.0 Hz, H-2, 6), 2.18 (4H, t, *J* = 7.0 Hz, H-3, 5). ^13^C NMR (100 MHz, DMSO-*d*_6_) *δ*_C_: 24.9 (q, C-8, 9), 28.8 (t, C-2, 6), 28.9 (s, C-4), 34.1 (t, C-3, 5), 175.0 (s, C-1, 7); positive ESI-MS *m*/*z* 211 [M+Na]^+^.

**Figure S1**. ^1^H NMR spectrum (600 MHz, CDCl_3_) of **1**


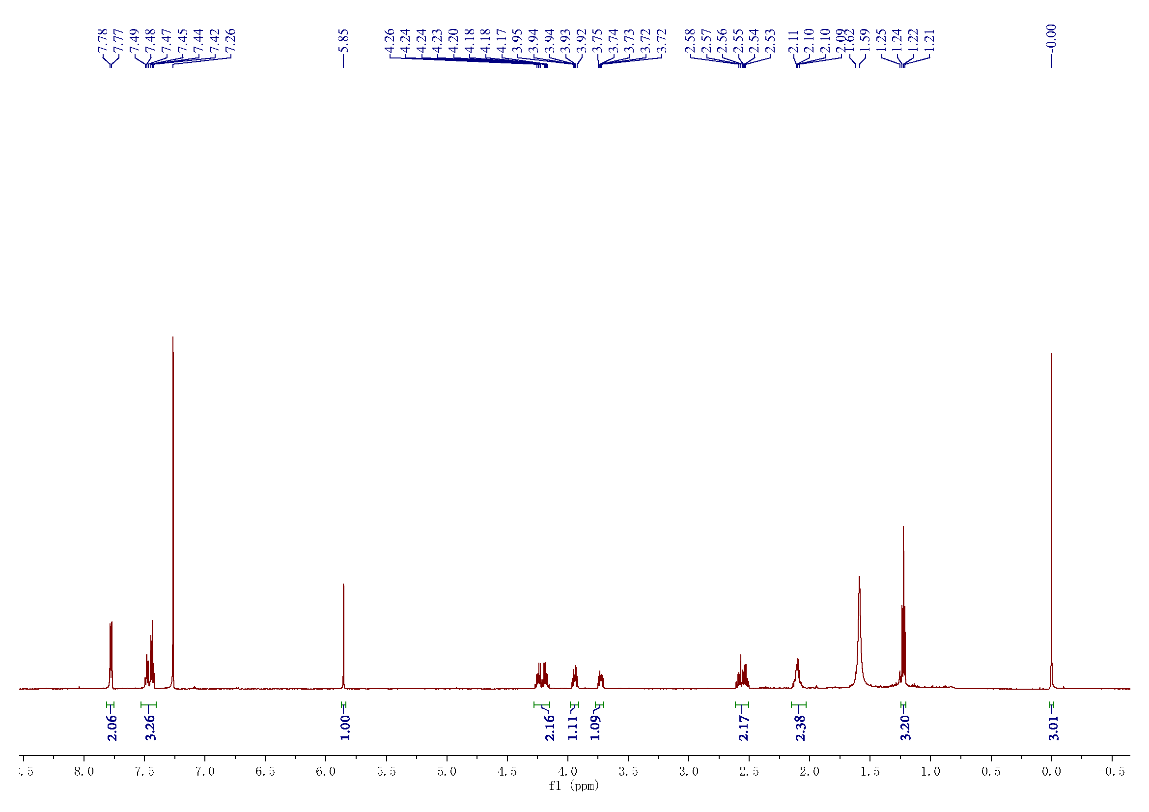


**Figure S2.** ^13^C NMR and DEPT spectrum (150 MHz, CDCl_3_) of **1**

**
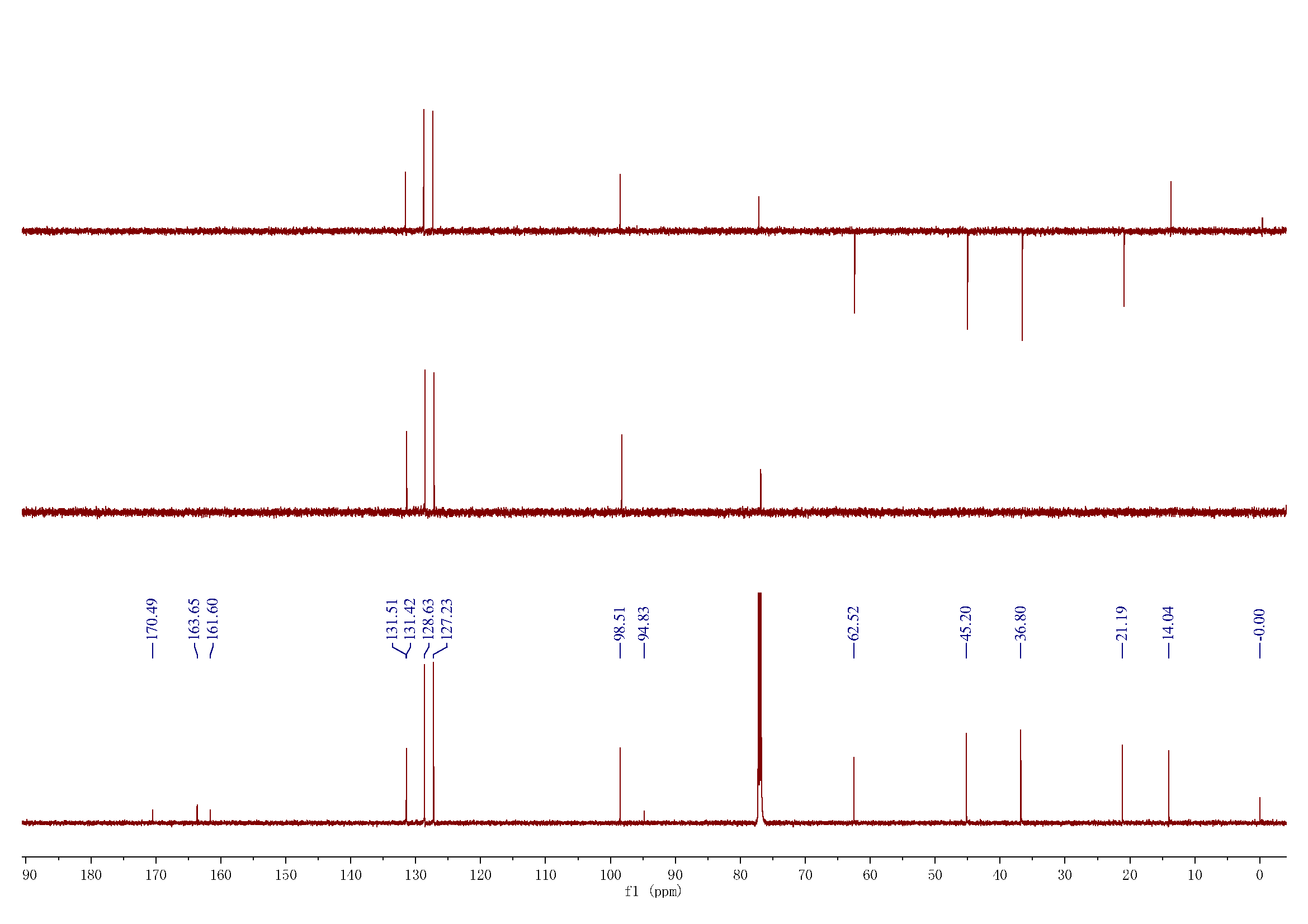
**

**Figure S3.** HSQC spectrum (600 MHz, CDCl_3_) of **1**


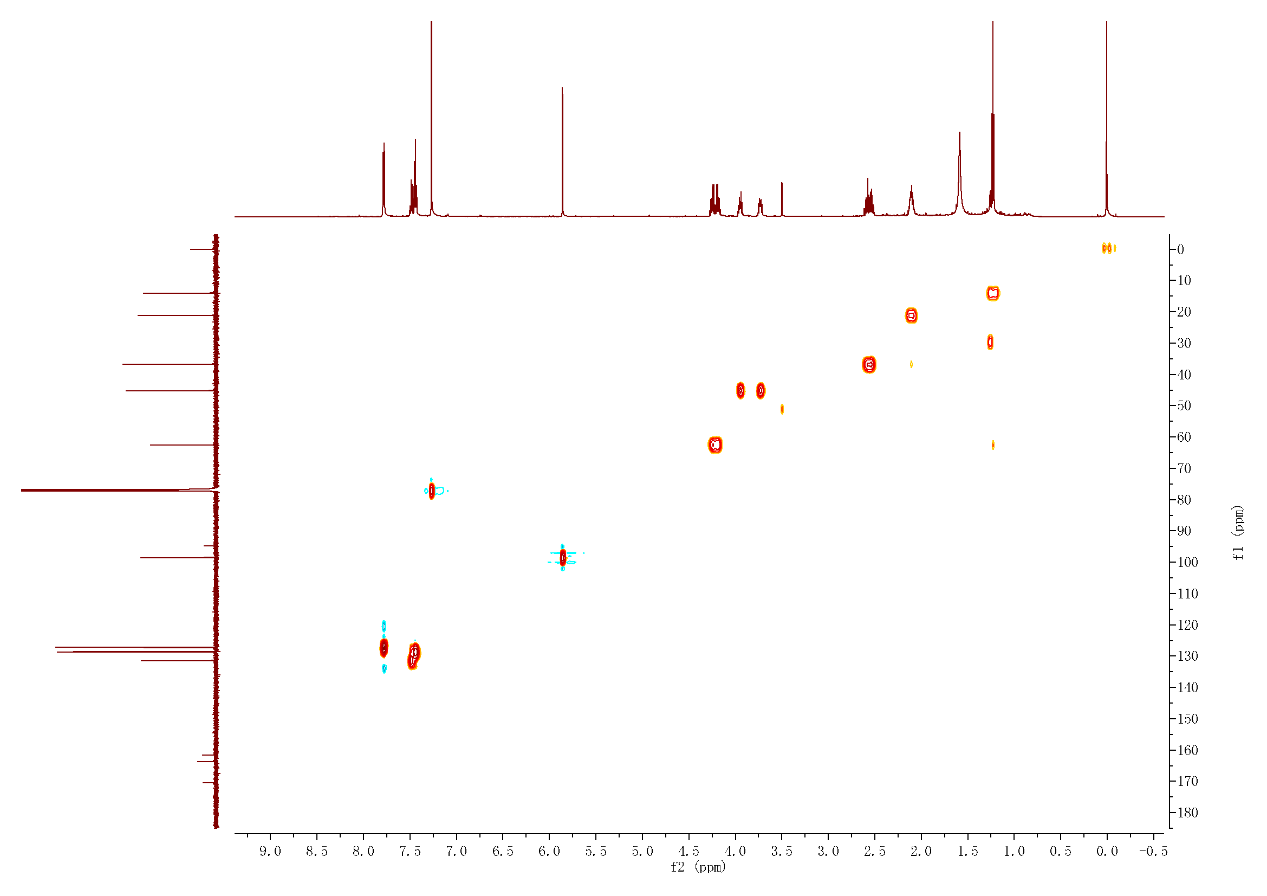


**Figure S4**. HMBC spectrum (600 MHz, CDCl_3_) of **1**


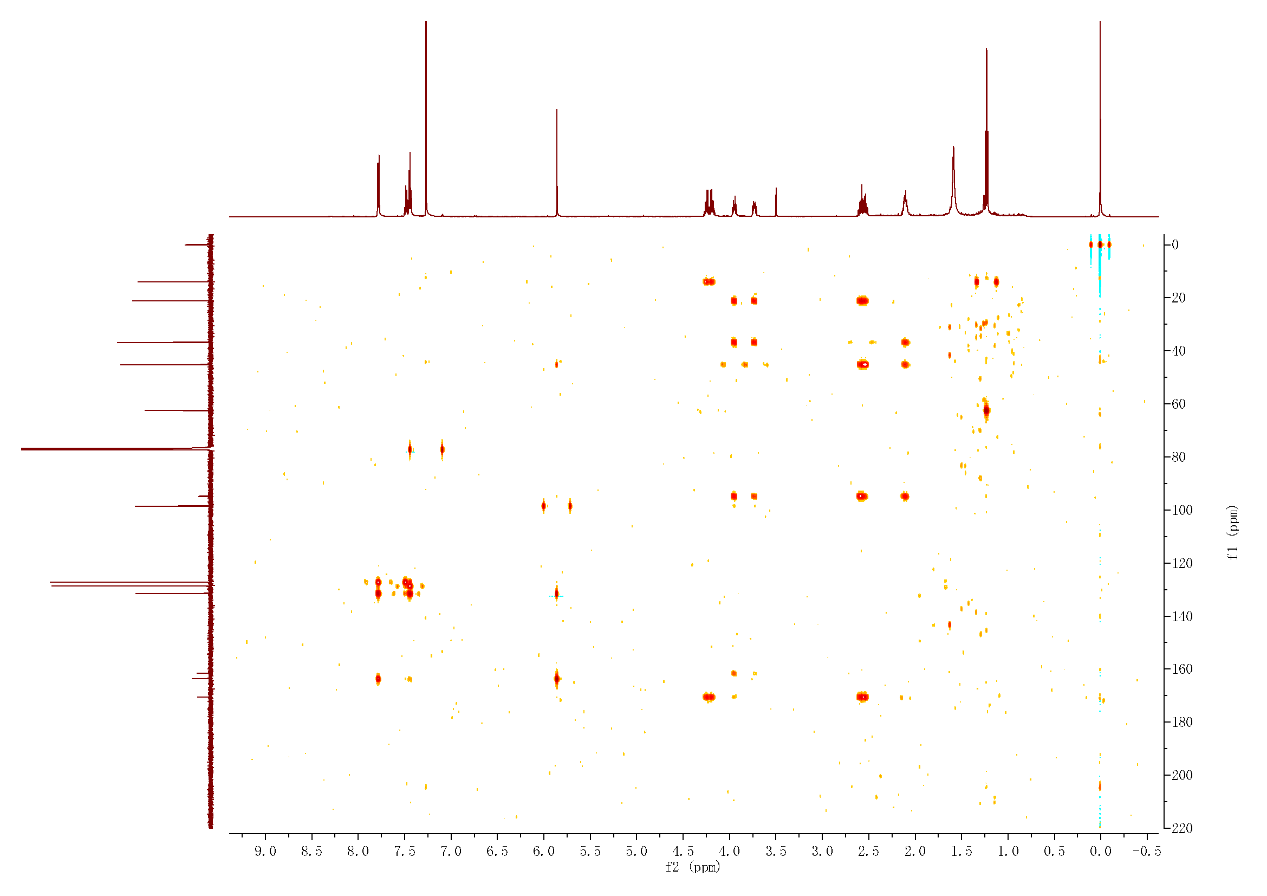


**Figure S5.** ^1^H-^1^H COSY spectrum (600 MHz, CDCl_3_) of **1**


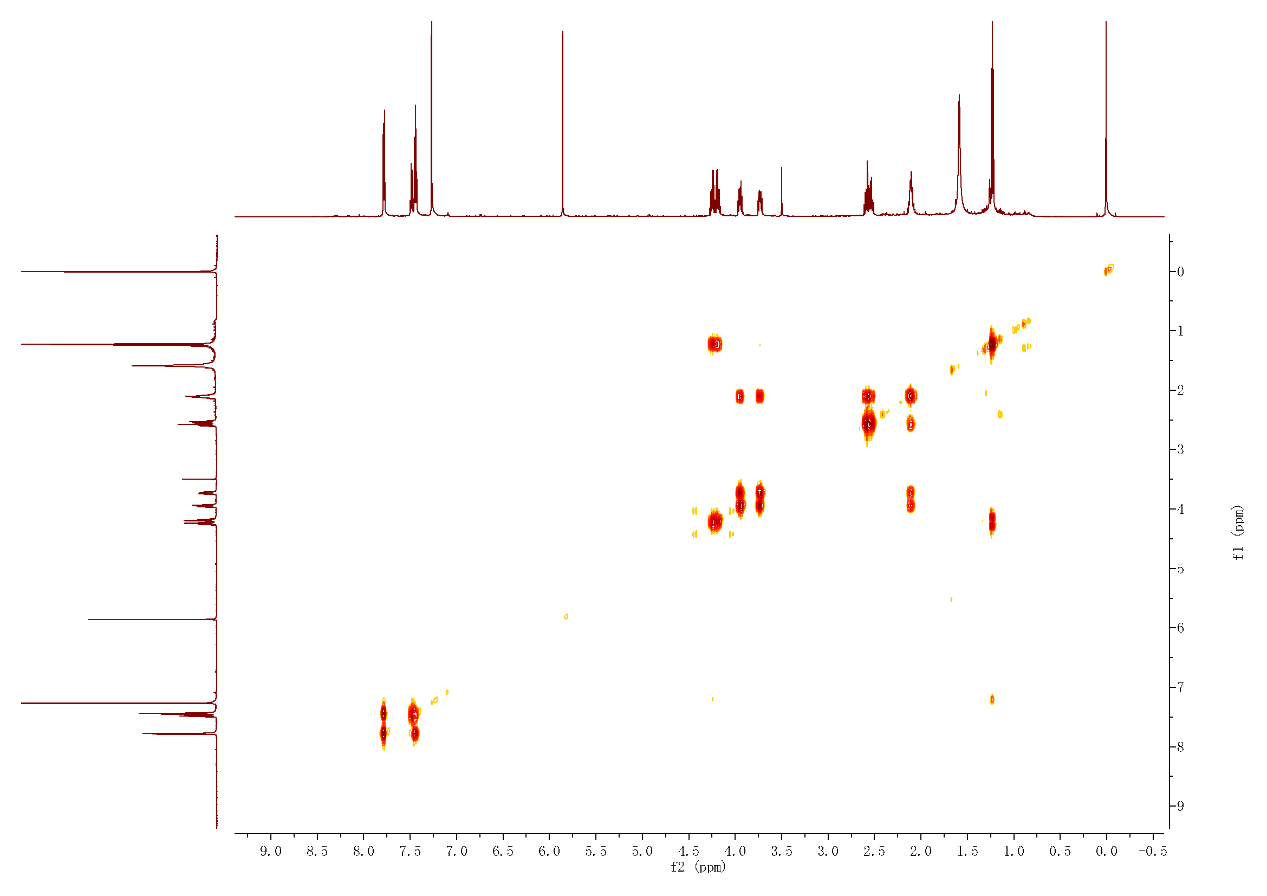


**Figure S6.** NOESY spectrum (600 MHz, CDCl_3_) of **1**


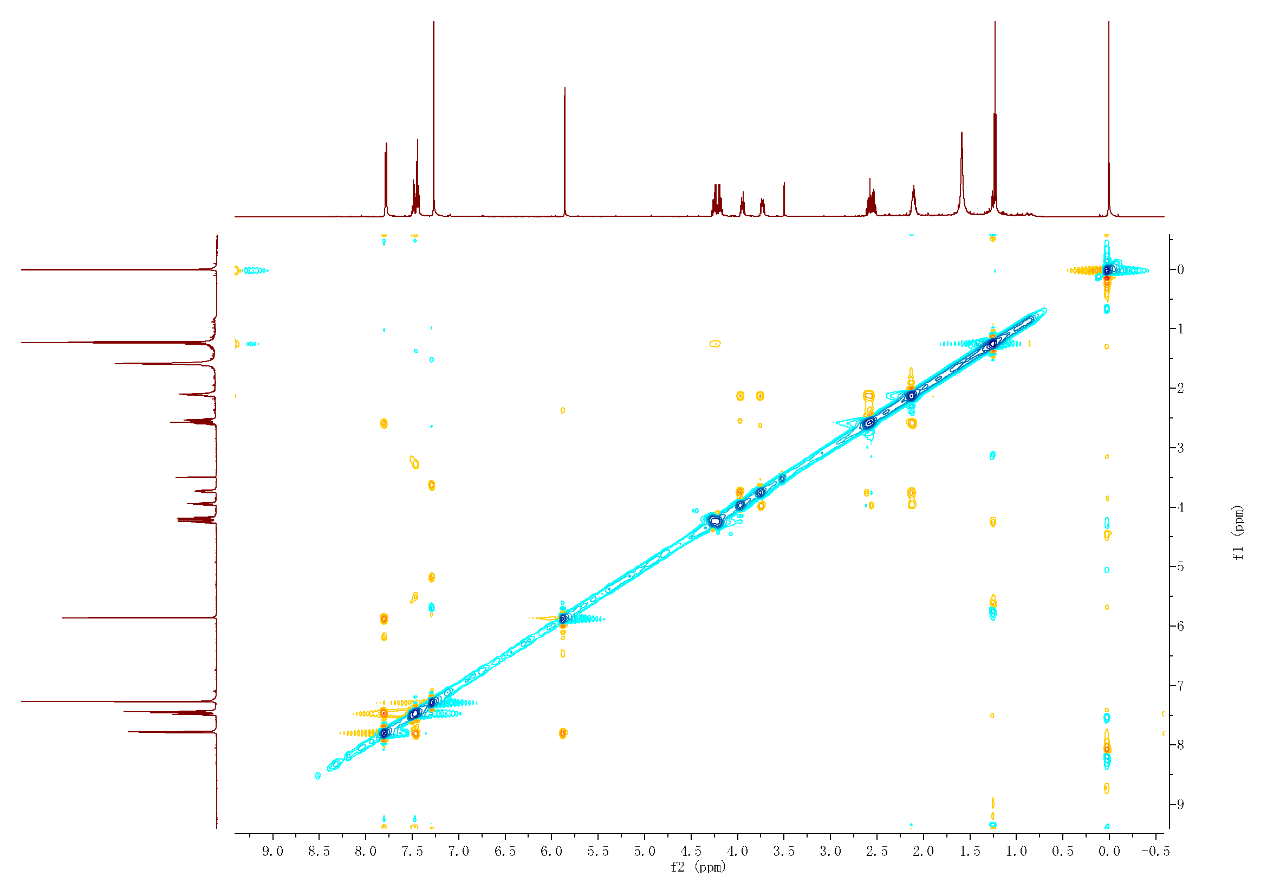


**Figure S7.** IR spectrum of **1**


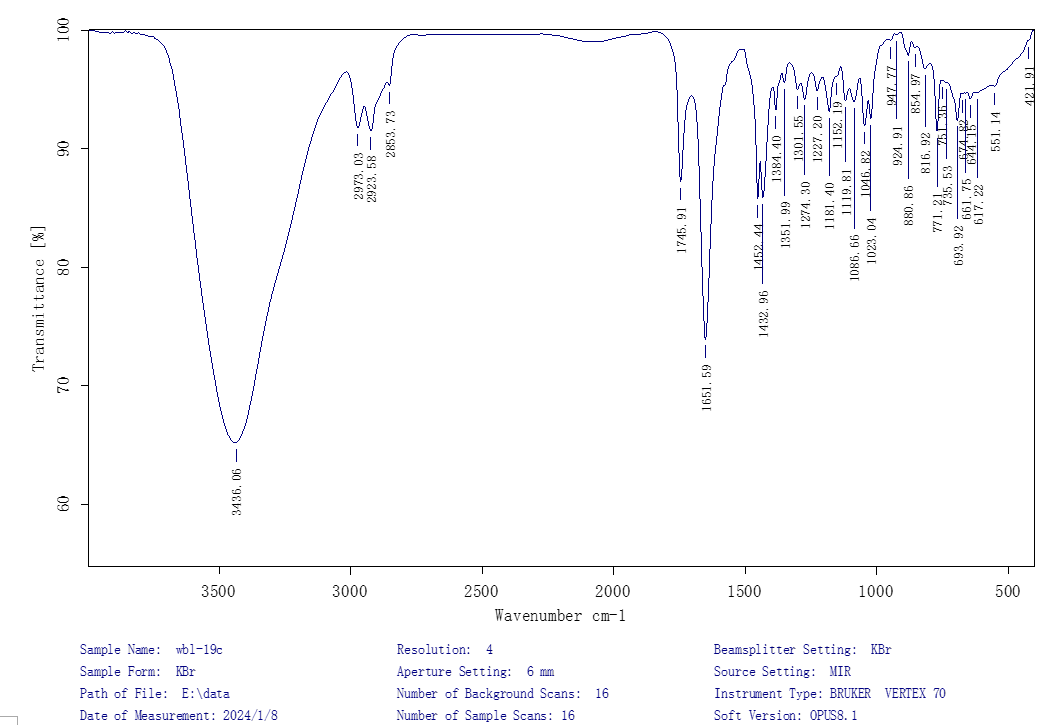


**Figure S8.** HR-ESIMS spectrum of **1**


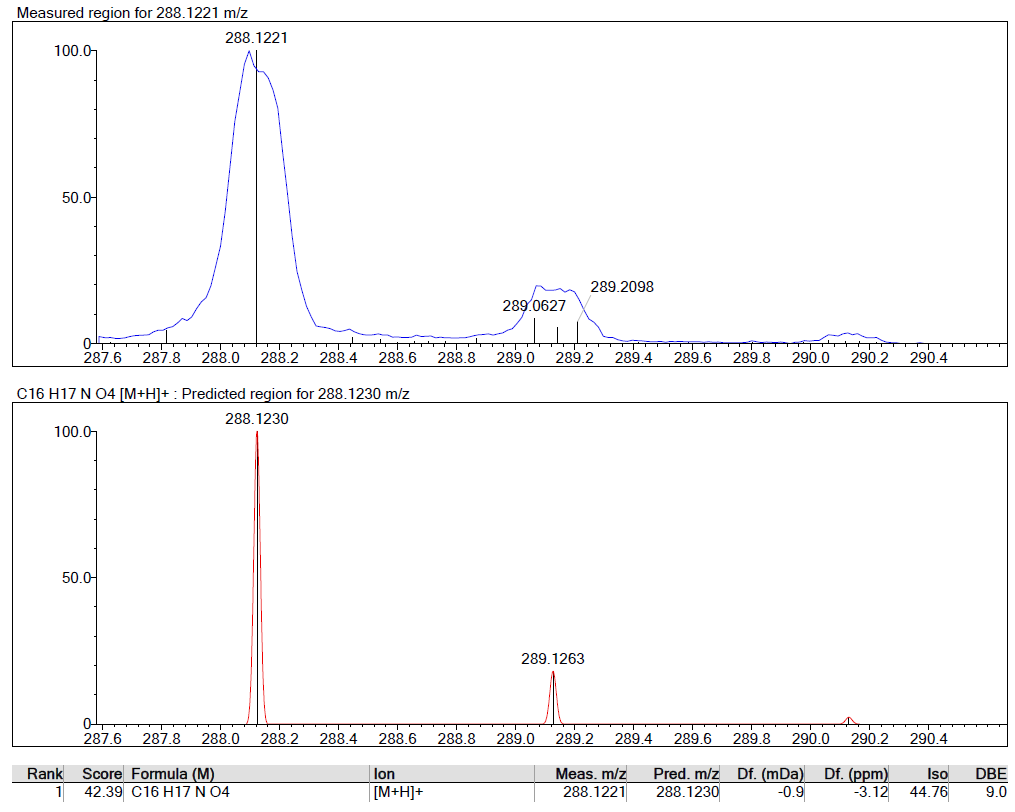


**Figure S9.** UV spectrum of compound **1**


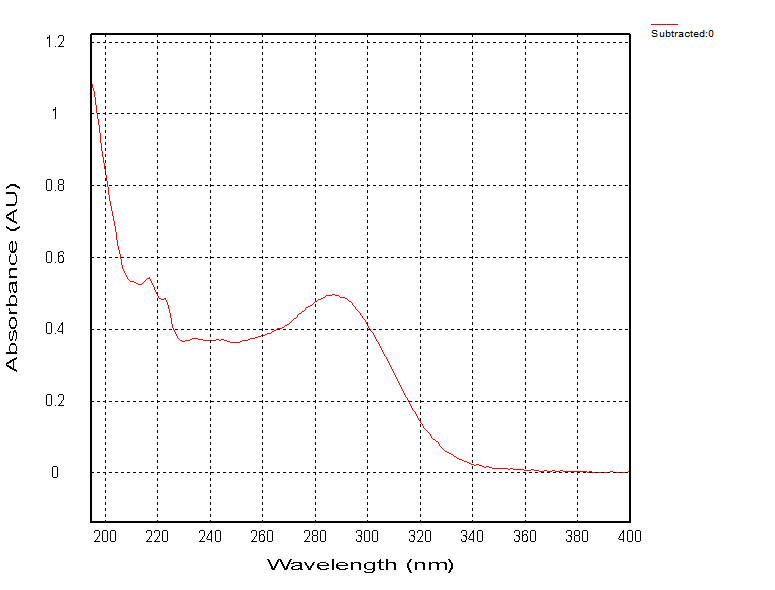


**Figure S10.** Circular dichroism spectrum of **1**

_
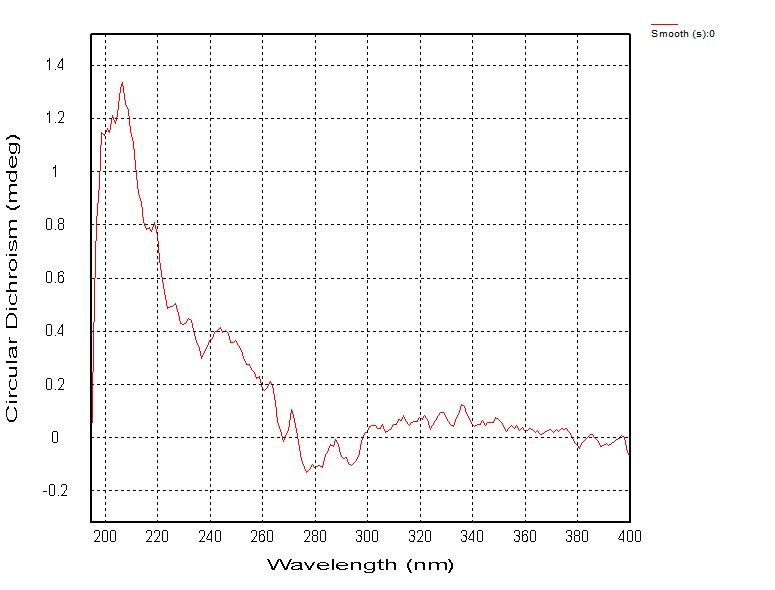
_

**Figure S11.** Specific rotation spectrum of **1**


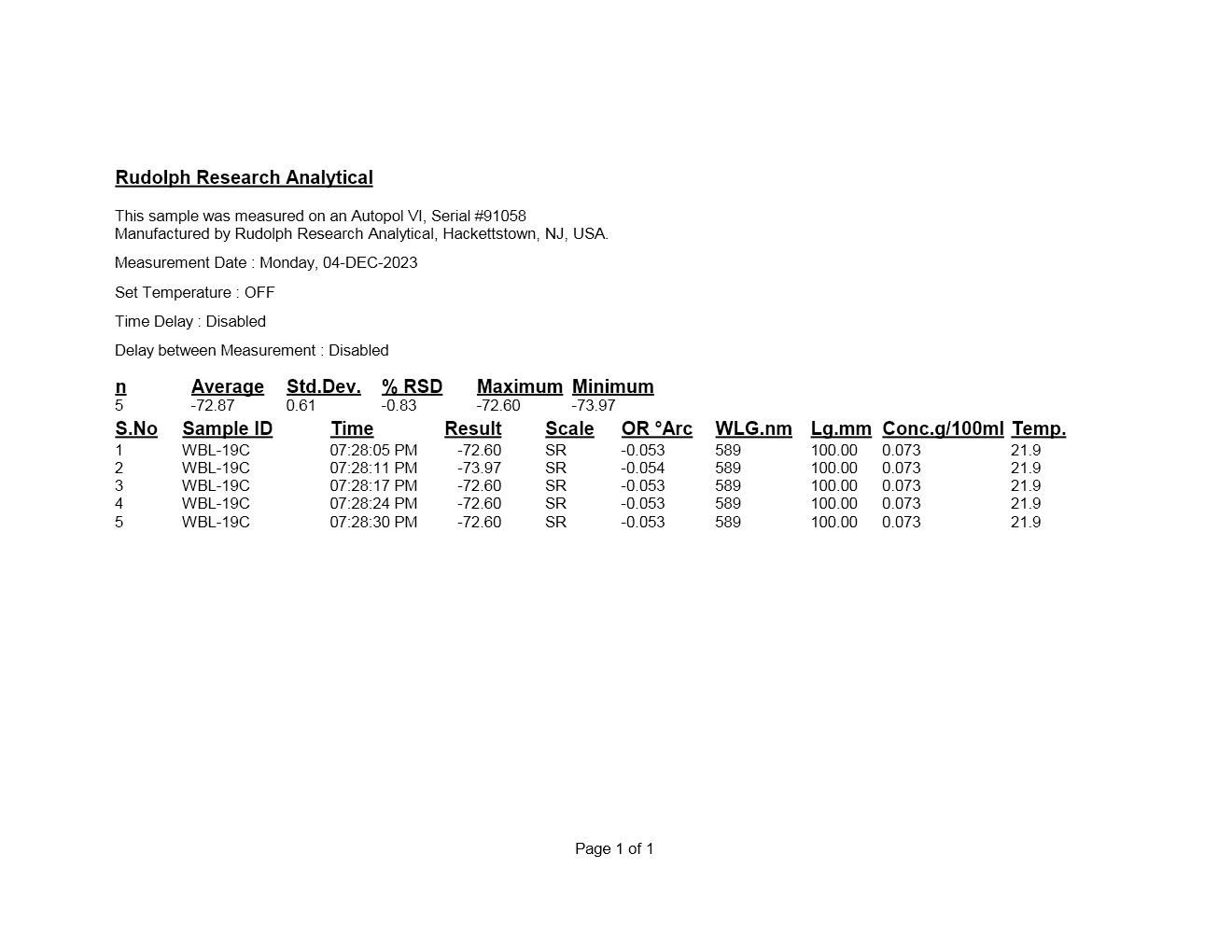


**Figure S12.** ^1^H NMR spectrum (600 MHz, CD_3_OD) of **2**


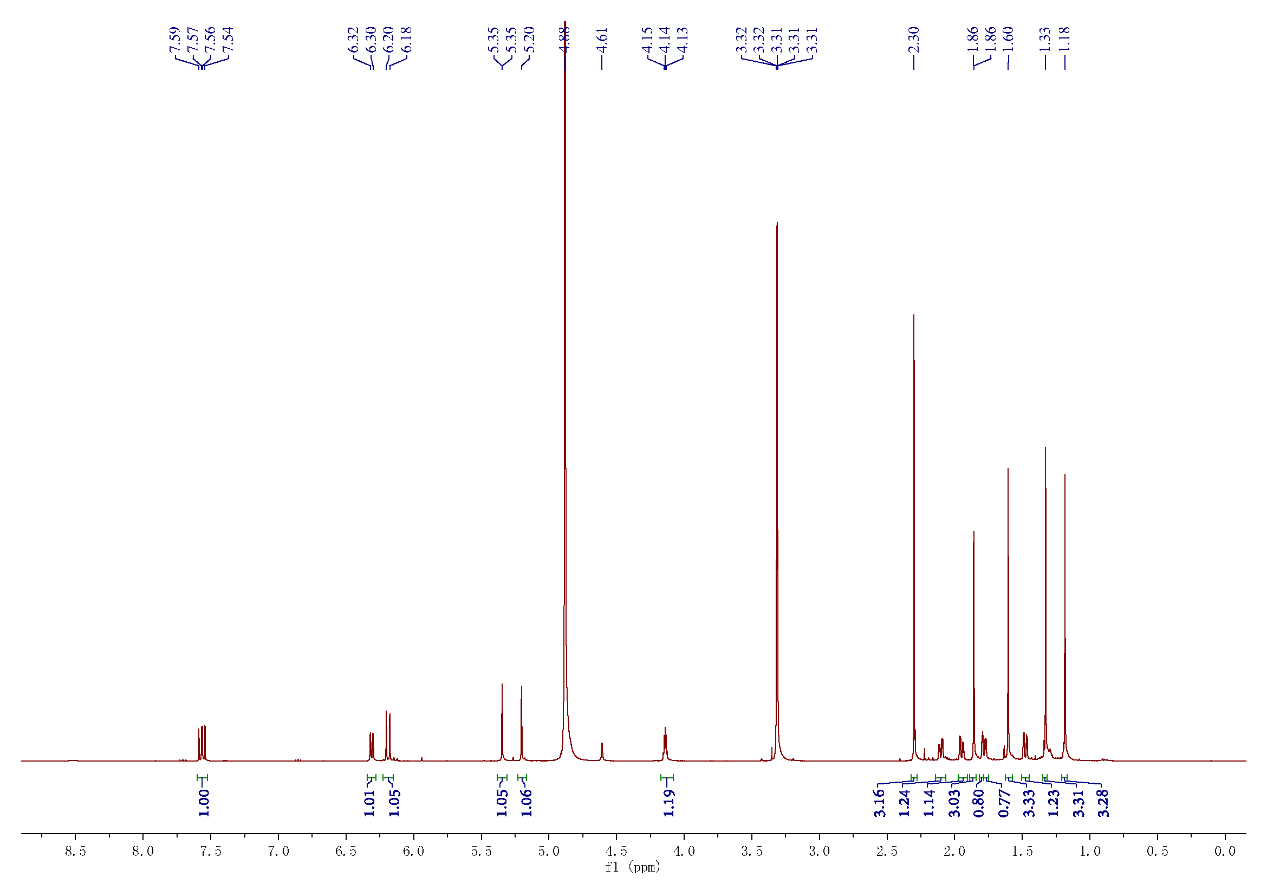


**Figure S13.** ^13^C NMR and DEPT spectrum (150 MHz, CD_3_OD) of **2**


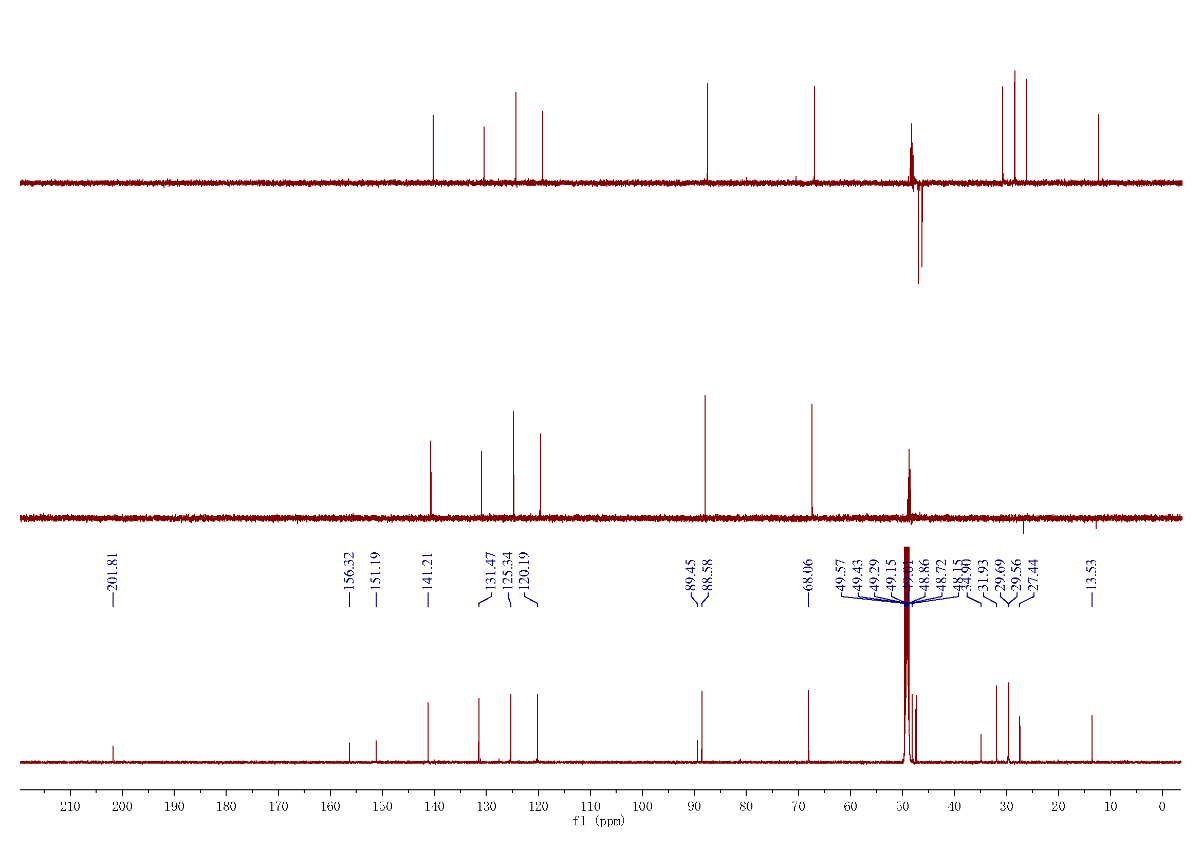


**Figure S14.** HSQC spectrum (600 MHz, CD_3_OD) of **2**


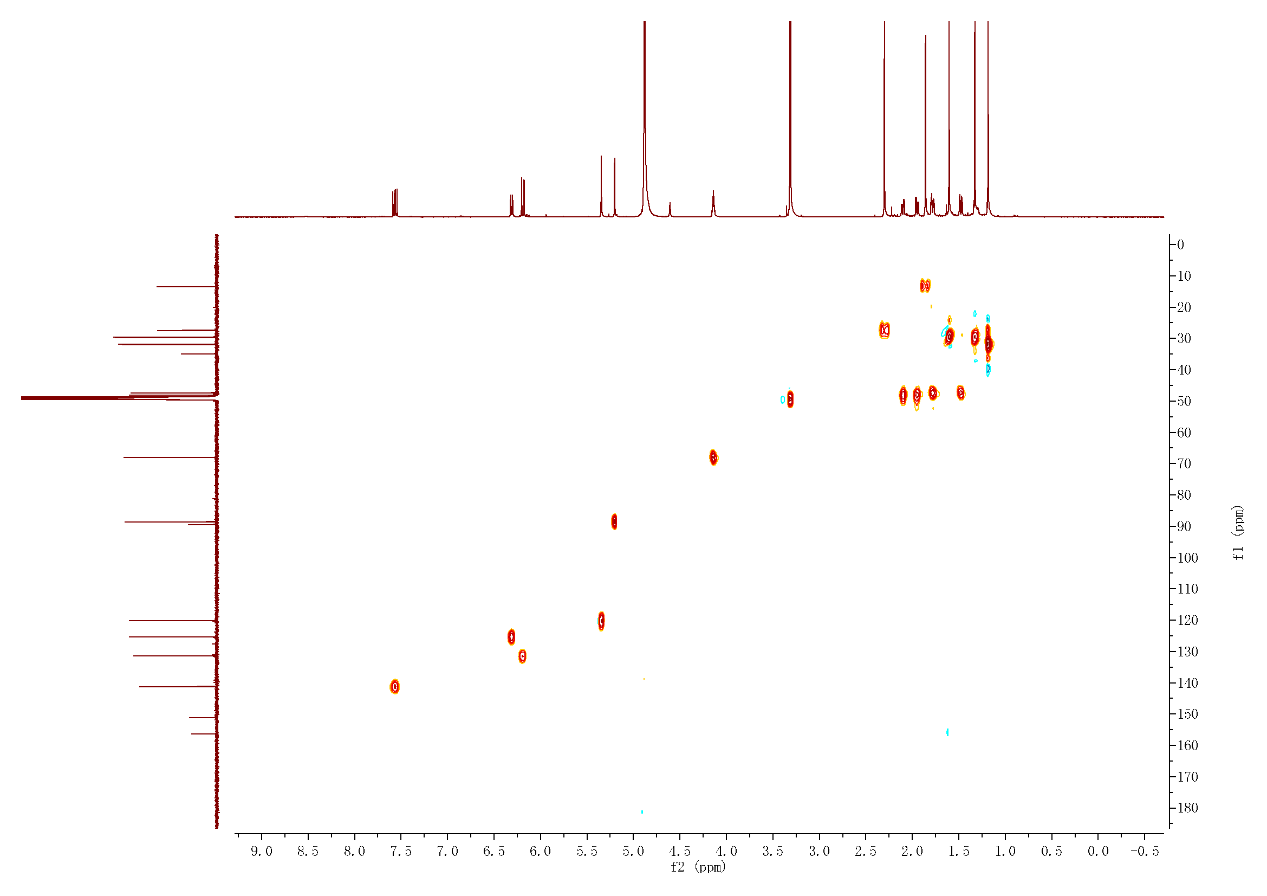


**Figure S15.** HMBC spectrum (600 MHz, CD_3_OD) of **2**


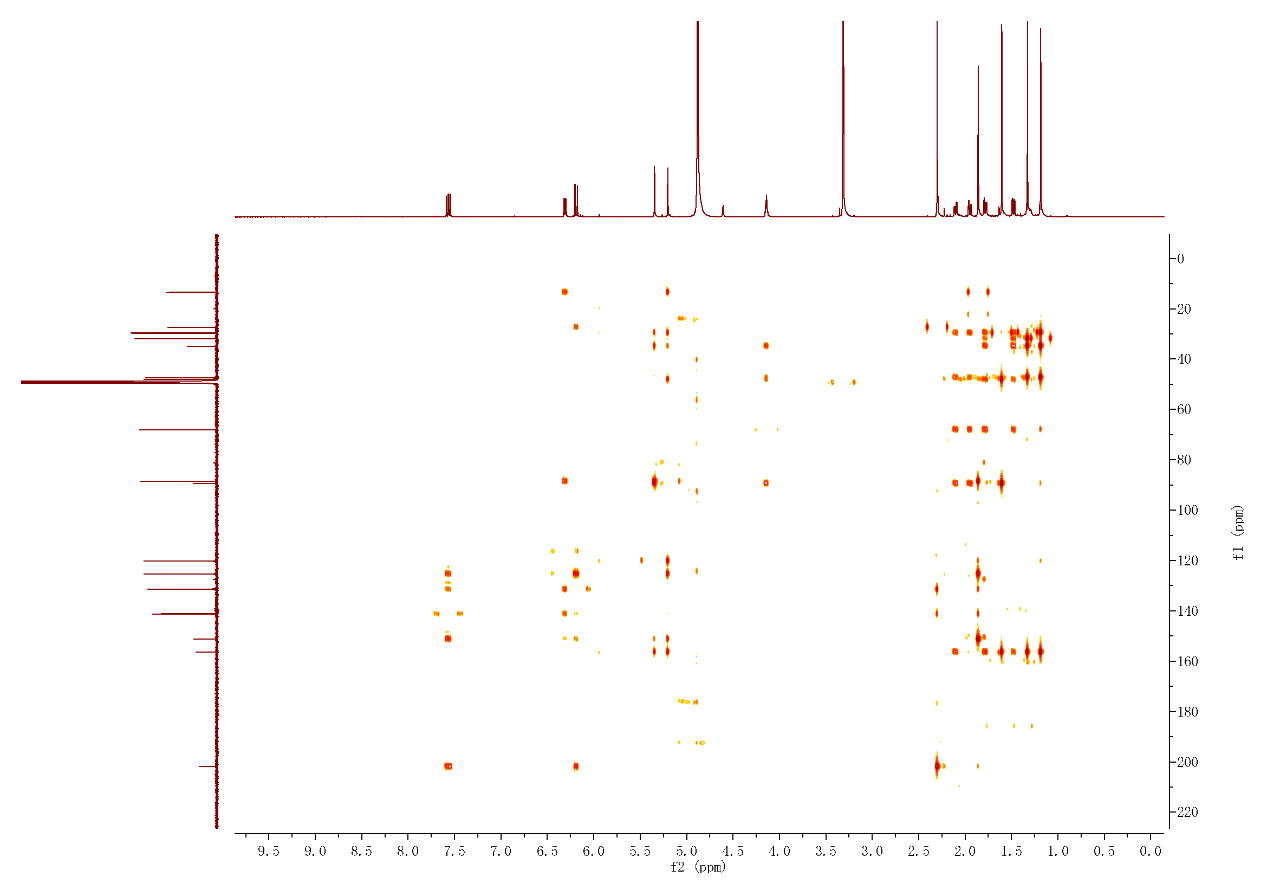


**Figure S16.** ^1^H-^1^H COSY spectrum (600 MHz, CD_3_OD) of **2**


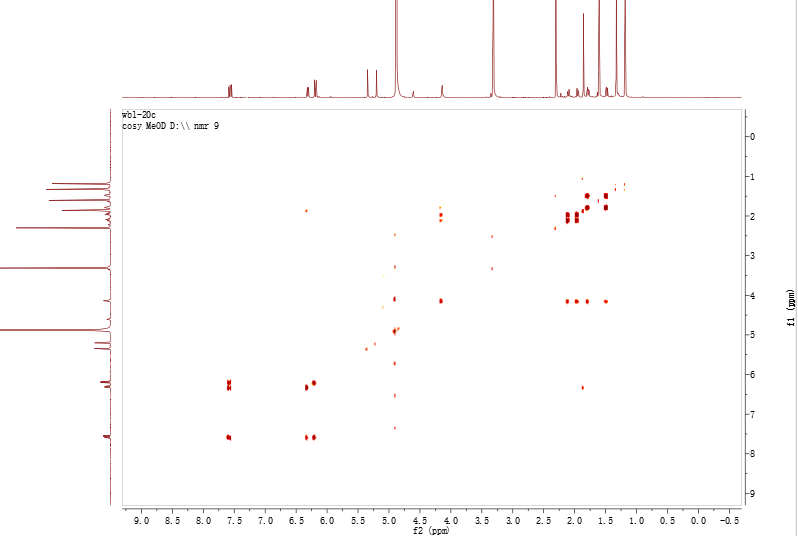


**Figure S17.** NOESY spectrum (600 MHz, CD_3_OD) of **2**
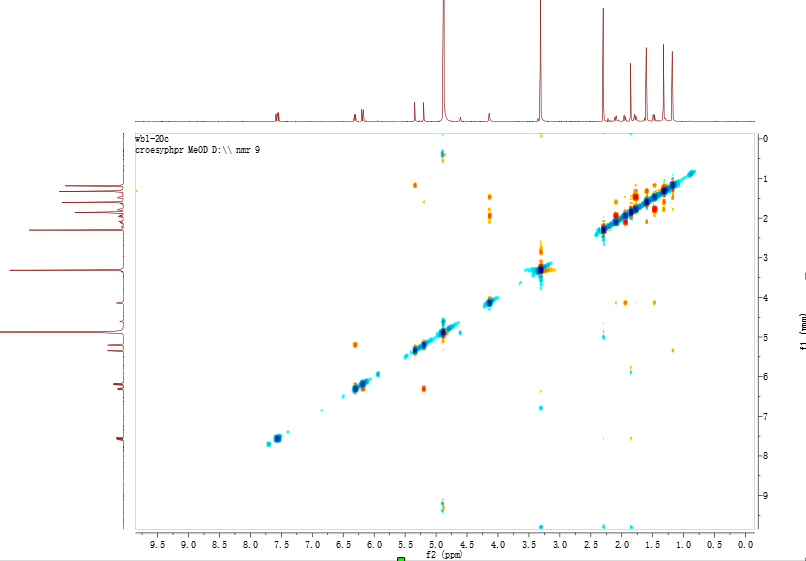


**Figure S18.** IR spectrum of **2**


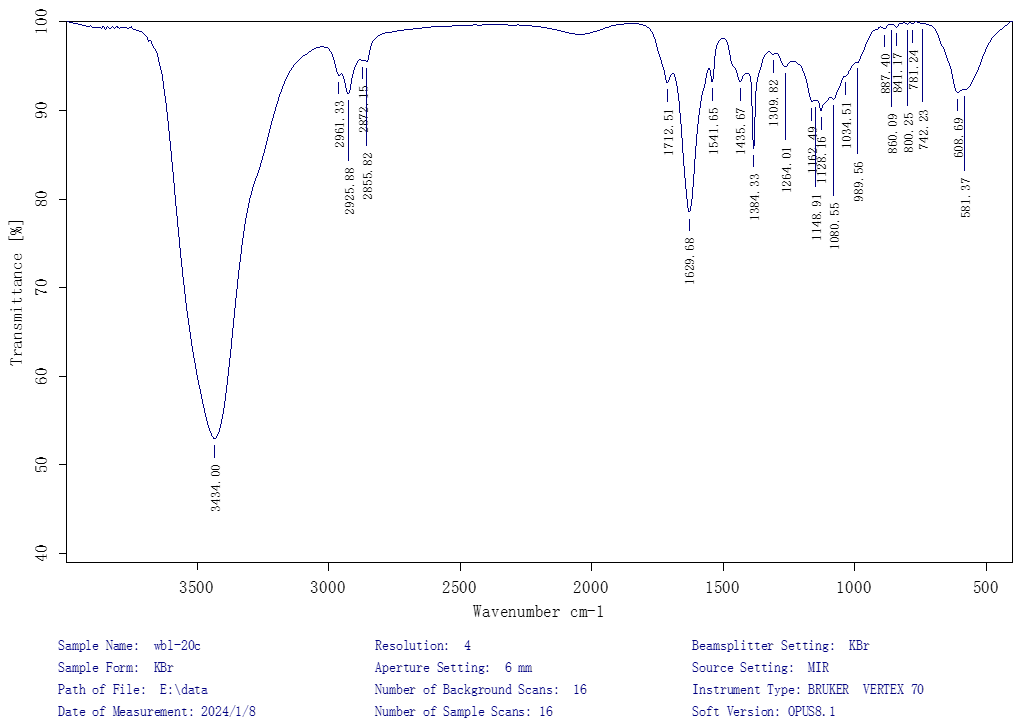


**Figure S19**. HRESIMS spectrum of **2**


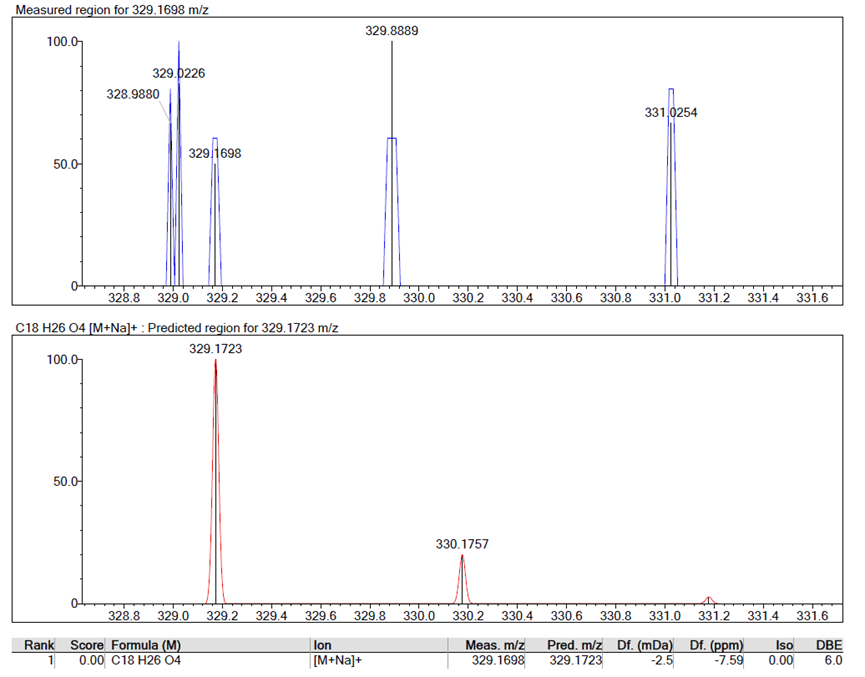


**Figure S20.** UV spectrum of compound **2**


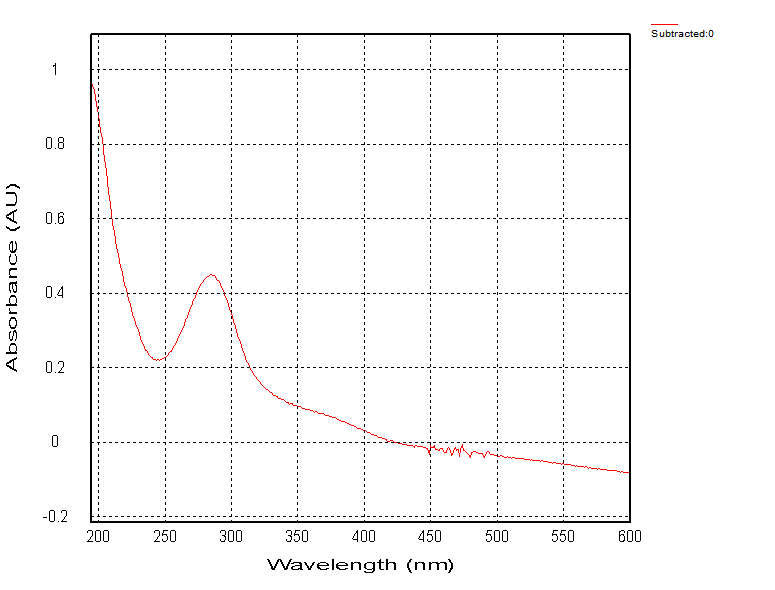


**Figure S21.** Circular dichroism spectrum of **2**


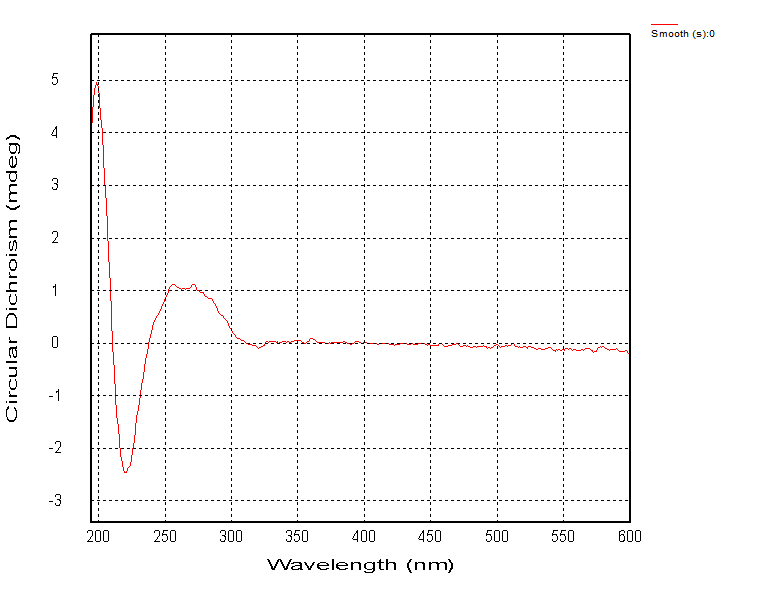


**ECD Calculation Details.**

The conformation search was performed by Spartan 14.0 software using Molecular Merck force field (MMFF). The appropriate low-energy conformers were selected and optimized in the gas phase using the Gaussian 16 program package. Further optimization and frequency analysis were carried out using density functional theory (DFT) at the B3LYP/6-31G(d,p) level. Solvent effects were accounted for by employing the conductor polarizable continuum model (CPCM). All conformers were utilized for calculating electronic circular dichroism (ECD) through the time-dependent density functional theory (TDDFT) method at the B3LYP/6-31G(d, p) level with the CPCM model in MeOH. The overall calculated ECD curves were generated by Boltzmann weighting the selected low-energy conformers using SpecDis 1.62 with σ ≈ 0.3 eV

**Plant material**

*L. barbarum* leaves were gathered from Zhongning country in P. R. China on May 10, 2016, kept dry, and ventilated. And the material was discerned by Dr. Jian-Fei Liu from LanZhou Institute of Chemical Physics, CAS. The voucher specimen (No. 20160510) has been stored in a dried environment of Kunming Institute of botany, Chinese Academy of Sciences.

**Extraction and purification**

The 14.0 kg of air-dried *L. barbarum* leaves were ground into powder and extracted four times at room temperature using 85% ethanol (each 80 L). A crude residue (3.0 kg) was obtained by concentrating the mixed extracts under lower pressure. After dissolving the crude residue in 5.0 L of water, it was extracted five times using ethyl acetate, yielding 780.0 g of ethyl acetate extract. Six fractions (Frs. A‒F) were obtained by subjecting this to silica gel column chromatography (Si CC) and eluting it gradually using a mixed solvent of MeOH−CHCl_3_ (0:100, 10:90, 20:80, 30:70, 40:60 and 50:50, *v*/*v*).

Fr. D (87.0 g) was separated *via* MCI gel CHP 20P CC, eluted with MeOH−H_2_O (30:70 to 100:0) to obtain Frs. D_1_‒D_4_. Fr. D_4_ was then purified on an Rp-C18 gel column, eluted with MeOH−H_2_O (50:50), yielding compound **32** (10.0 g).

Fr. E (99.0 g) was separated using an MCI gel CHP 20P CC with a MeOH−H_2_O (30:70 to 100:0) gradient, resulting in four fractions (Frs. E_1_‒E_4_). Fr. E_1_ (5.0 g) underwent Si CC (CHCl_3_−acetone, 20:1 to 10:1), isolating compound **5** (5.0 mg). Further purification involved Si CC (petroleum ether−EtOAc, 3:1 to 2:1) and Sephadex LH-20 (MeOH), yielding compounds **24** (20.0 mg), **13** (5.0 mg), and **14** (20.0 mg).

Fr. E_2_ was purified on an Rp-C18 gel column with MeOH−H_2_O (30:70 to 100:0), and Sephadex LH-20 (MeOH), producing compounds **28** (1.0 g), **23** (20.0 mg), **25** (100.0 mg), **9** (5.0 mg), **27** (10.0 mg), **28** (5.0 mg), and **33** (4.1 mg). The remaining portion was processed by HPLC to yield compounds **5** (40.0 mg, *t_R_* = 24 min; CH_3_CN−H_2_O, 20:80) and **10** (40.0 mg, *t_R_* = 27 min; CH_3_CN−H_2_O, 20:80). Fr. E_4_ was purified *via* HPLC to yield compounds **2** (4.2 mg, *t_R_* = 35 min; CH3CN−H2O, 45:55), **4** (6.8 mg, *t_R_* = 31 min; CH_3_CN−H_2_O, 15:85), and **7** (2.0 mg, *t_R_* = 14 min; CH_3_CN−H_2_O, 12:88).

Fraction F (80.0 g) was separated *via* MCI gel CHP 20P CC with a MeOH−H_2_O (20:80 to 100:0) gradient, resulting in three fractions (Fr. F_1_‒F_3_). Fr. F_1.1_ (6.5 g) underwent Si CC (petroleum ether−acetone, 4:1 to 1:1) and Sephadex LH-20 (MeOH), isolating compounds **21** (2.0 g), **22** (10.0 mg), and **29** (4.5 mg). Subfraction Fr._1.2_ was further purified using Si CC (CHCl_3_−acetone, 30:1) to yield compounds **6** (10.0 mg), **20** (25.0 mg) and compound **3** (13.0 mg). HPLC was used to isolate compounds **1** (5.2 mg, *t_R_* = 21 min; CH_3_CN−H_2_O, 29:71) and **17** (30.0 mg, retention time = 24 min; CH_3_CN−H_2_O, 20:80) from Fr. F_1.3_. Fr. F_2_ (15.0 g) was chromatographed on Si CC (petroleum ether−EtOAc, 6:1 to 1:1) to obtain compounds **15** (2.0 mg), **16** (4.2 mg), **19** (5.0 mg), **26** (16.0 mg), followed by Sephadex LH-20 (MeOH) to yield compounds and **31** (4.1 mg), **18** (50.0 mg), **8** (2.0 mg), and **34** (100.0 mg).

**Table S1.** The cell viabilities of 34 compounds on MH7A by LPS induction

| No. | Group | Cell viability (%) | P value |
| --- | --- | --- | --- |
| Screening 1 | Control | 100.00±0.50 | - |
|  | Model | 123.02±1.35 | 0.000 |
|  | Compound **20** | 108.69±1.09 | 0.000 |
|  | Compound **21** | 99.58±0.78 | 0.000 |
| Screening 2 | Control | 100.00±0.92 | - |
|  | Model | 129.56±0.87 | 0.000 |
|  | Compound **4** | 103.32±2.96 | 0.000 |
|  | Compound **8** | 105.86±2.10 | 0.000 |
| Screening 3 | Control | 100.00±0.04 | - |
|  | Model | 115.98±0.94 | 0.000 |
|  | Compound **6** | 104.28±1.04 | 0.000 |
|  | Compound **29** | 112.46±1.06 | 0.009 |
| Screening 4 | Control | 100.00±0.20 |  |
|  | Model | 118.07±2.32 | 0.000 |
|  | Compound **5** | 116.99±2.34 | 0.601 |
|  | Compound **30** | 98.69±0.68 | 0.000 |
| Screening 5 | Control | 100.00±0.45 | - |
|  | Model | 116.47±2.64 | 0.000 |
|  | Compound **2** | 103.46±0.77 | 0.001 |
|  | Compound **9** | 102.50±1.11 | 0.001 |
| Screening 6 | Control | 100.00±0.75 | - |
|  | Model | 117.83±1.61 | 0.000 |
|  | Compound **12** | 99.88±0.57 | 0.000 |
|  | Compound **13** | 100.51±0.73 | 0.000 |
| Screening 7 | Control | 100.00±0.55 | - |
|  | Model | 115.44±1.06 | 0.000 |
|  | Compound **15** | 114.34±1.44 | 0.430 |
|  | Compound **23** | 96.54±2.07 | 0.000 |
| Screening 8 | Control | 100.00±0.81 | - |
|  | Model | 115.24±3.68 | 0.011 |
|  | Compound **14** | 94.84±1.80 | 0.000 |
|  | Compound **17** | 95.57±1.92 | 0.001 |
| Screening 9 | Control | 100.00±1.26 | - |
|  | Model | 114.09±2.18 | 0.003 |
|  | Compound **31** | 95.32±2.70 | 0.000 |
| Screening 10 | Control | 100.00±1.05 | - |
|  | Model | 116.92±3.27 | 0.001 |
|  | Compound **24** | 107.52±1.33 | 0.009 |
|  | Compound **25** | 117.68±1.11 | 0.723 |
| Screening 11 | Control | 100.00±3.14 | - |
|  | Model | 124.72±0.86 | 0.000 |
|  | Compound **11** | 98.44±0.78 | 0.000 |
|  | Compound **19** | 107.27±2.59 | 0.000 |
| Screening 12 | Control | 100.00±1.67 | - |
|  | Model | 128.68±0.72 | 0.000 |
|  | Compound **10** | 105.63±0.66 | 0.000 |
|  | Compound **16** | 109.24±1.27 | 0.000 |
| Screening 13 | Control | 100.00±1.04 | - |
|  | Model | 114.36±2.41 | 0.000 |
|  | Compound **3** | 106.42±2.83 | 0.014 |
|  | Compound **7** | 114.85±1.80 | 0.784 |
|  | Compound **18** | 98.47±0.90 | 0.000 |
| Screening 14 | Control | 100.00±1.18 | - |
|  | Model | 121.72±2.51 | 0.000 |
|  | Compound **34** | 115.31±6.02 | 0.164 |
| Screening 15 | Control | 100.00±3.47 | - |
|  | Model | 112.91±2.30 | 0.005 |
|  | Compound **1** | 98.72±1.62 | 0.000 |
| Screening 16 | Control | 100.00±4.29 | - |
|  | Model | 114.54±0.90 | 0.004 |
|  | Compound **27** | 115.66±2.16 | 0.453 |
|  | Compound **32** | 96.45±1.74 | 0.000 |
|  | Compound **33** | 112.37±1.89 | 0.147 |
| Screening 17 | Control | 100.00±2.07 | - |
|  | Model | 121.01±2.18 | 0.000 |
|  | Compound **26** | 116.76±2.30 | 0.420 |
|  | Compound **22** | 104.95±4.77 | 0.110 |
|  | Compound **28** | 118.49±1.83 | 0.200 |

**Table S2.** The effect of 24 compounds on the production of NO in LPS-induced MH7A

| NO. | Group | NO (μM) | P value |
| --- | --- | --- | --- |
| Screening 1 | Control | 0.92±0.06 | - |
|  | Model | 1.36±0.04 | 0.000 |
|  | Compound **2** | 1.00±0.10 | 0.004 |
| Screening 2 | Control | 0.84±0.05 | - |
|  | Model | 1.20±0.04 | 0.000 |
|  | Compound **4** | 1.15±0.01 | 0.183 |
|  | Compound **12** | 1.07±0.03 | 0.018 |
|  | Compound **14** | 0.92±0.07 | 0.004 |
| Screening 3 | Control | 0.72±0.02 | - |
|  | Model | 1.29±0.03 | 0.000 |
|  | Compound **19** | 1.01±0.08 | 0.006 |
| Screening 4 | Control | 0.92±0.06 | - |
|  | Model | 1.26±0.04 | 0.001 |
|  | Compound **16** | 1.00±0.10 | 0.014 |
| Screening 5 | Control | 0.77±0.03 | - |
|  | Model | 1.31±0.02 | 0.000 |
|  | Compound **9** | 1.30±0.03 | 0.590 |
|  | Compound **13** | 1.25±0.03 | 0.081 |
|  | Compound **21** | 1.14±0.02 | 0.000 |
| Screening 6 | Control | 0.79±0.01 | - |
|  | Model | 1.29±0.03 | 0.000 |
|  | Compound **29** | 1.07±0.03 | 0.008 |
|  | Compound **30** | 1.24±0.03 | 0.192 |
|  | Compound **32** | 1.25±0.05 | 0.367 |
| Screening 7 | Control | 0.79±0.01 | - |
|  | Model | 1.27±0.02 | 0.000 |
|  | Compound **6** | 1.16±0.03 | 0.007 |
|  | Compound **24** | 1.23±0.02 | 0.093 |
| Screening 8 | Control | 0.75±0.06 | - |
|  | Model | 1.17±0.03 | 0.000 |
|  | Compound **3** | 1.01±0.05 | 0.038 |
|  | Compound **17** | 0.85±0.01 | 0.000 |
|  | Compound **18** | 0.82±0.04 | 0.000 |
| Screening 10 | Control | 0.92±0.02 | - |
|  | Model | 1.34±0.01 | 0.000 |
|  | Compound **11** | 1.06±0.07 | 0.003 |
|  | Compound **20** | 1.31±0.05 | 0.361 |
| Screening 11 | Control | 0.75±0.04 | - |
|  | Model | 1.20±0.04 | 0.000 |
|  | Compound **8** | 0.92±0.04 | 0.001 |
|  | Compound **31** | 0.92±0.02 | 0.000 |
| Screening 12 | Control | 0.73±0.03 | - |
|  | Model | 1.40±0.05 | 0.000 |
|  | Compound **1** | 0.75±0.04 | 0.000 |
|  | Compound **10** | 1.02±0.02 | 0.000 |
|  | Compound **23** | 1.38±0.01 | 0.689 |

**Table S3.** The effect of 24 compounds on the release of LDH in LPS-induced MH7A

| No. | Group | LDH (U/gprot) | P value |
| --- | --- | --- | --- |
| Screening 1 | Control | 1.29±0.03 | - |
|  | Model | 1.60±0.02 | 0.000 |
|  | Compound **3** | 1.35±0.02 | 0.000 |
|  | Compound **17** | 1.38±0.01 | 0.000 |
|  | Compound **18** | 1.30±0.01 | 0.000 |
| Screening 2 | Control | 0.92±0.02 | - |
|  | Model | 1.80±0.04 | 0.000 |
|  | Compound **8** | 1.28±0.04 | 0.000 |
| Screening 3 | Control | 1.11±0.02 | - |
|  | Model | 1.71±0.02 | 0.000 |
|  | Compound **10** | 1.38±0.06 | 0.001 |
| Screening 4 | Control | 1.24±0.02 | - |
|  | Model | 1.44±0.03 | 0.000 |
|  | Compound **14** | 1.29±0.02 | 0.002 |
| Screening 5 | Control | 1.13±0.04 | - |
|  | Model | 1.63±0.03 | 0.000 |
|  | Compound **1** | 1.10±0.01 | 0.000 |
|  | Compound **2** | 1.26±0.05 | 0.000 |
|  | Compound **4** | 1.56±0.05 | 0.118 |
|  | Compound **19** | 1.34±0.03 | 0.000 |
| Screening 6 | Control | 1.20±0.06 | - |
|  | Model | 1.38±0.01 | 0.010 |
|  | Compound **12** | 1.33±0.03 | 0.139 |
|  | Compound **16** | 1.35±0.04 | 0.315 |
| Screening 7 | Control | 1.29±0.05 | - |
|  | Model | 1.49±0.04 | 0.005 |
|  | Compound **11** | 1.42±0.03 | 0.060 |
|  | Compound **30** | 1.42±0.03 | 0.080 |
|  | Compound **32** | 1.40±0.01 | 0.021 |
| Screening 8 | Control | 0.92±0.06 | - |
|  | Model | 1.39±0.07 | 0.000 |
|  | Compound **6** | 1.26±0.03 | 0.044 |
|  | Compound **13** | 1.32±0.03 | 0.217 |
|  | Compound **29** | 1.20±0.03 | 0.013 |
| Screening 9 | Control | 1.27±0.03 | - |
|  | Model | 1.62±0.04 | 0.000 |
|  | Compound **20** | 1.56±0.01 | 0.056 |
|  | Compound **21** | 1.58±0.01 | 0.115 |
|  | Compound **23** | 1.57±0.06 | 0.305 |
|  | Compound **24** | 1.56±0.03 | 0.100 |
| Screening 10 | Control | 1.28±0.01 | - |
|  | Model | 1.41±0.02 | 0.000 |
|  | Compound **9** | 1.28±0.04 | 0.004 |
|  | Compound **31** | 1.33±0.01 | 0.002 |

**Table S4.** The summary of effective compounds on both NO and LDH

| Compounds | NO inhibitory rate (%) | LDH inhibitory rate (%) |
| --- | --- | --- |
| **1** | 46.7 | 32.8 |
| **2** | 26.8 | 22.8 |
| **3** | 10.2 | 16.0 |
| **6** | 8.8 | 9.5 |
| **8** | 23.4 | 28.6 |
| **10** | 26.9 | 19.3 |
| **14** | 23.6 | 10.4 |
| **17** | 26.9 | 14.1 |
| **18** | 29.6 | 18.7 |
| **19** | 21.6 | 17.6 |
| **29** | 16.9 | 13.7 |
| **31** | 3.1 | 6.0 |
